# Supplementary material for: Approaches to the Synthesis of Dicarboxylic Derivatives of Bis(pyrazol-1-yl)alkanes
Source: Molecules. 2021 Jan 14;26(2):413. doi: 10.3390/molecules26020413 (PMC7829949; doi:10.3390/molecules26020413)
Supplement: Supplementary file 1 [file molecules-26-00413-s001.pdf]

Supplementary materials

Approaches to the synthesis of dicarboxylic derivatives of bis(pyrazol-1-yl)alkanes

Nikita P. Burlutskiy[1], Andrei S. Potapov [2,*]

[1] Kizhner Research Center, National Research Tomsk Polytechnic University, 30 Lenin Ave., 634050 Tomsk, Russia; npb1@tpu.ru

[2] Nikolaev Institute of Inorganic Chemistry, Siberian Branch of the Russian Academy of Sciences, 3 Lavrentiev Ave., 630090 Novosibirsk, Russia; potapov@niic.nsc.ru

* Correspondence: potapov@niic.nsc.ru

IR spectra of bis(pyrazol-1-yl)alkane derivatives

Compound 1b

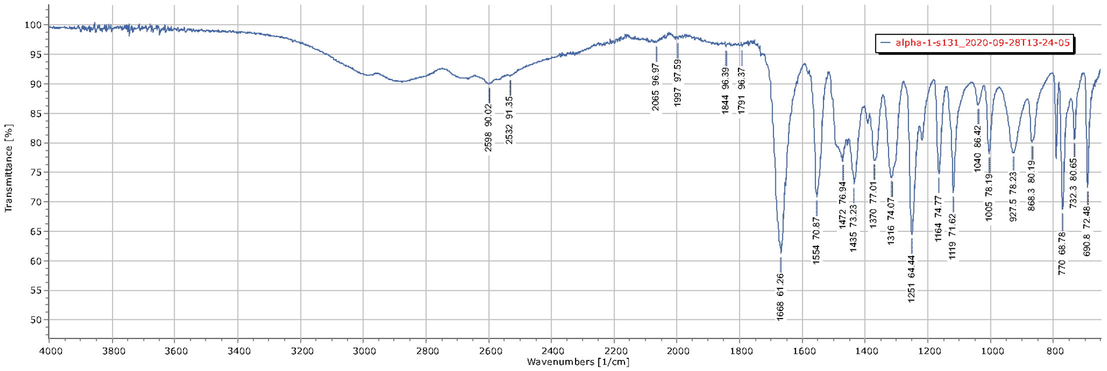

Compound 3b

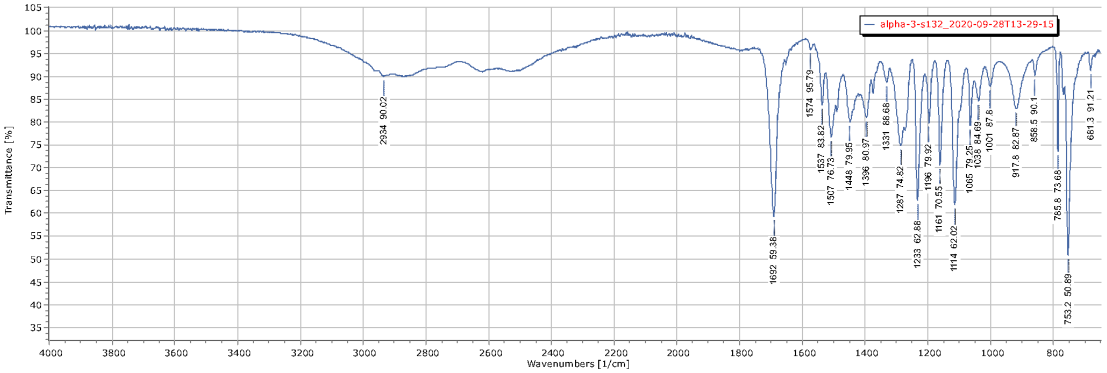

Compound 4b

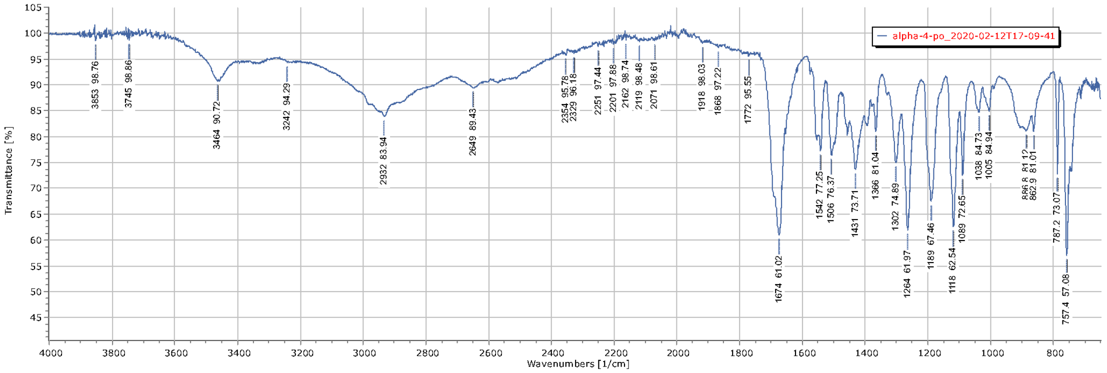

Compound 5b

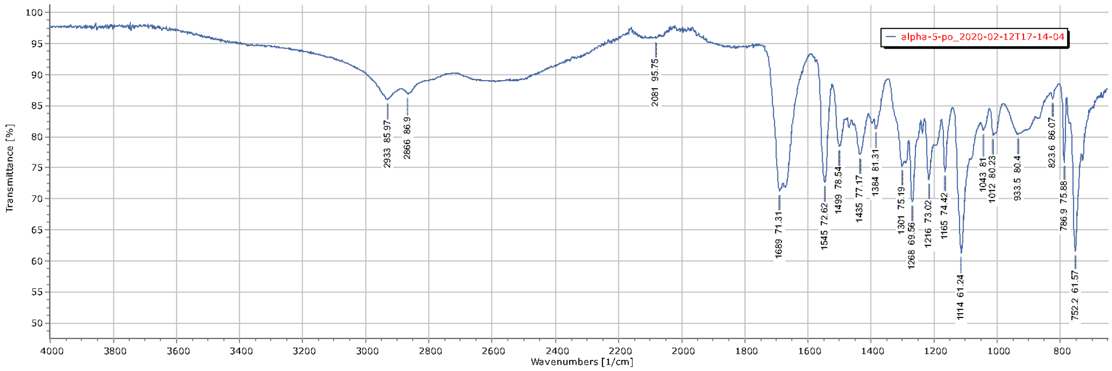

Compound 6b

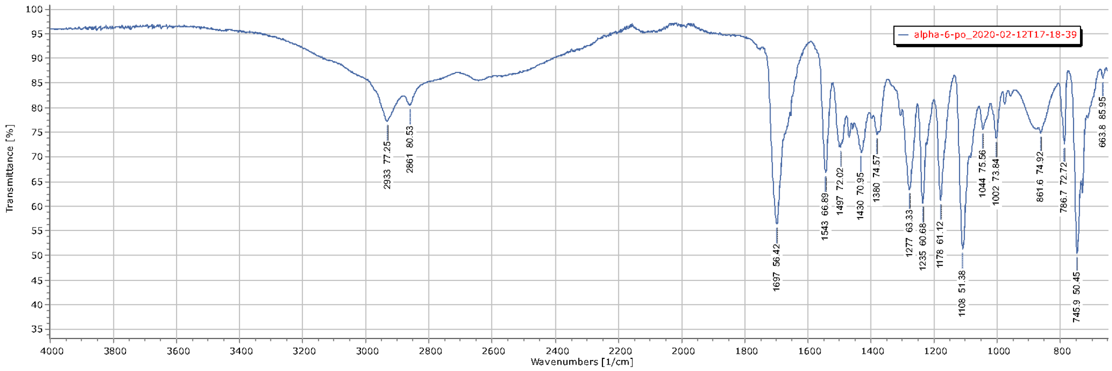

Compound 4c

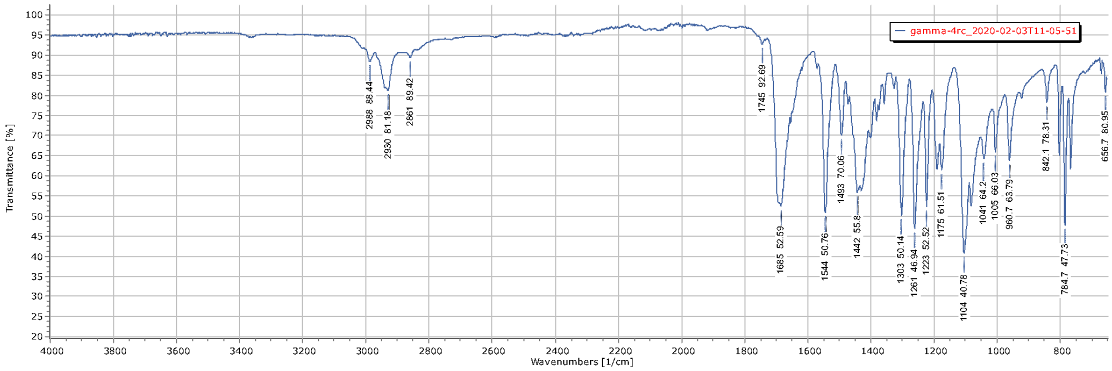

Compound 5c

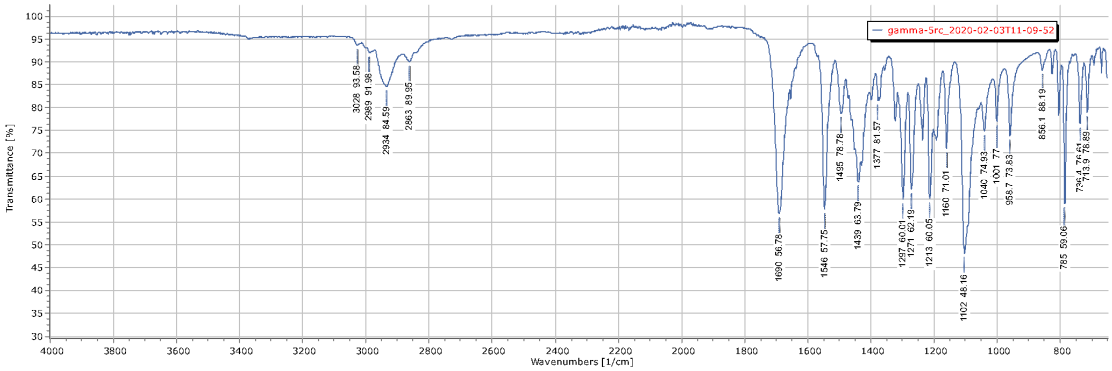

Compound 6c

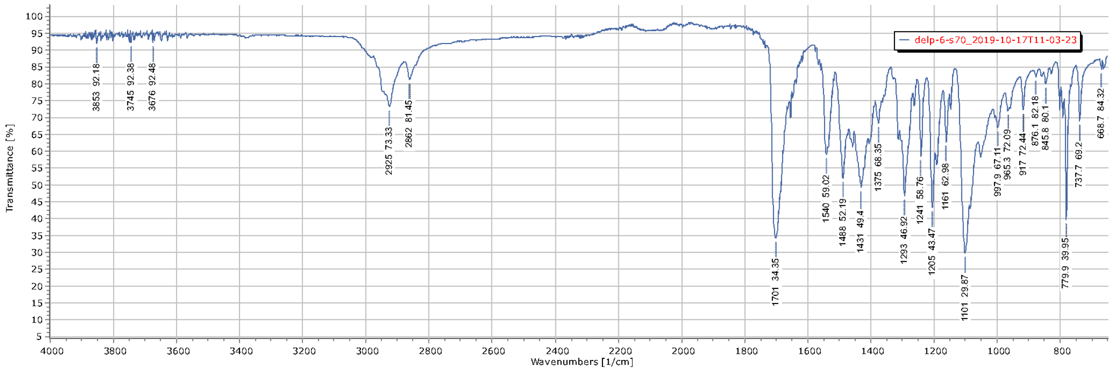

Compound 1e

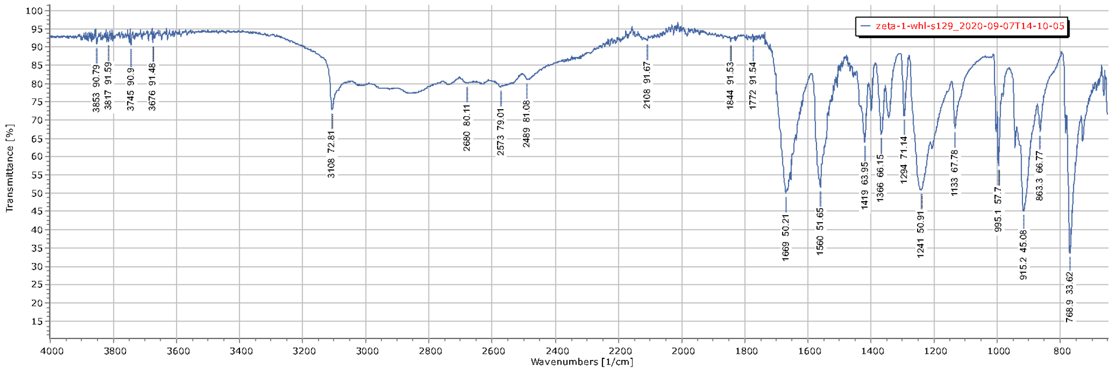

Compound 3e

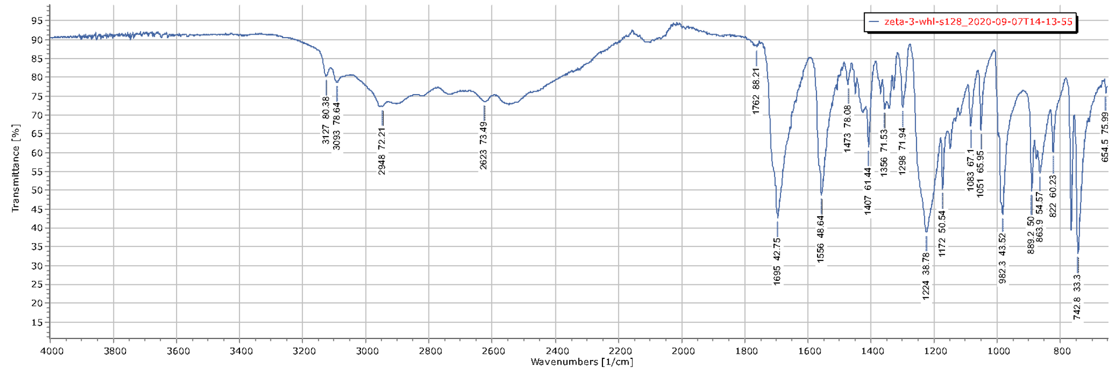

Compound 4e

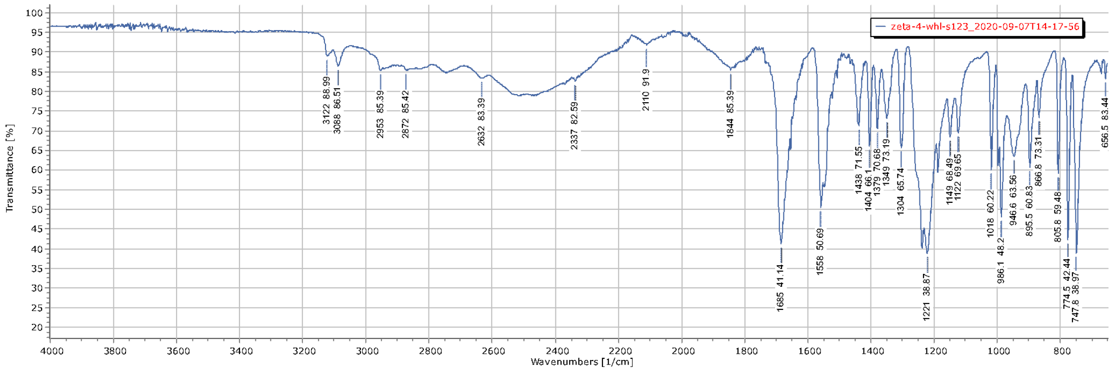

Compound 5e

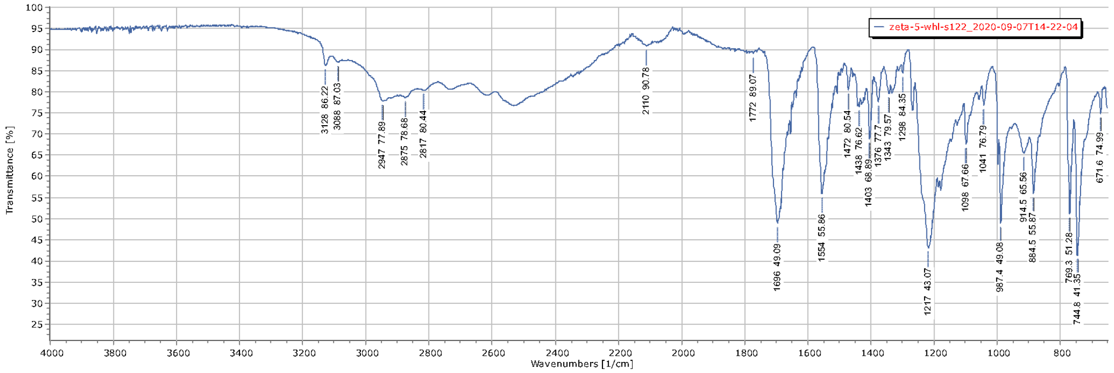

Compound 6e

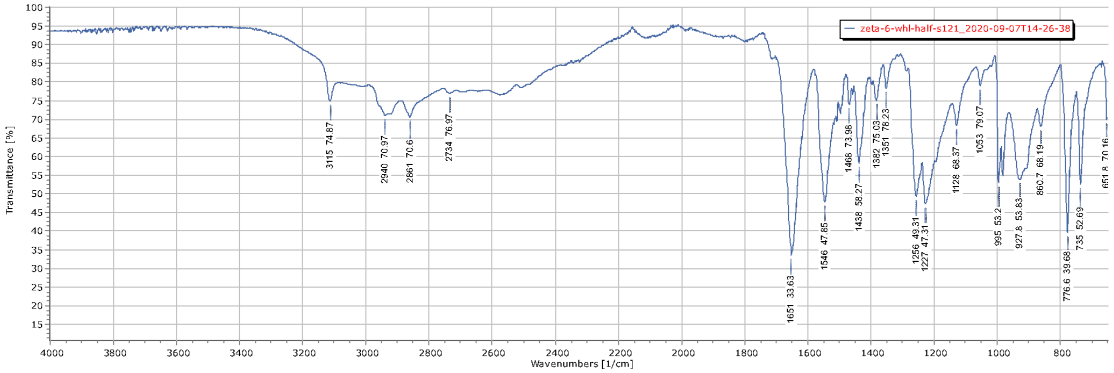

Compound 1f

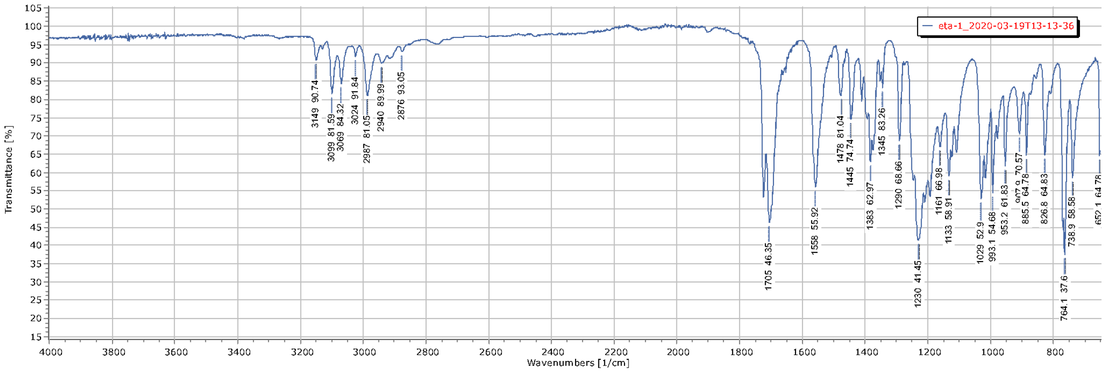

Compound 3f

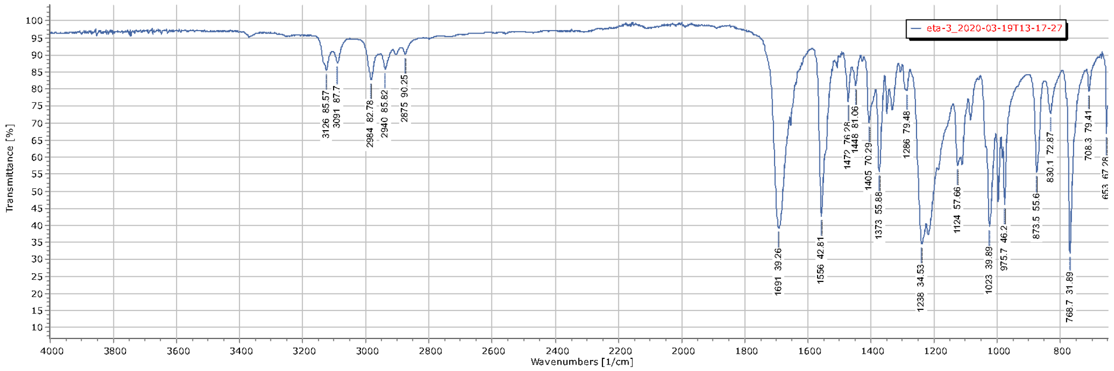

Compound 4f

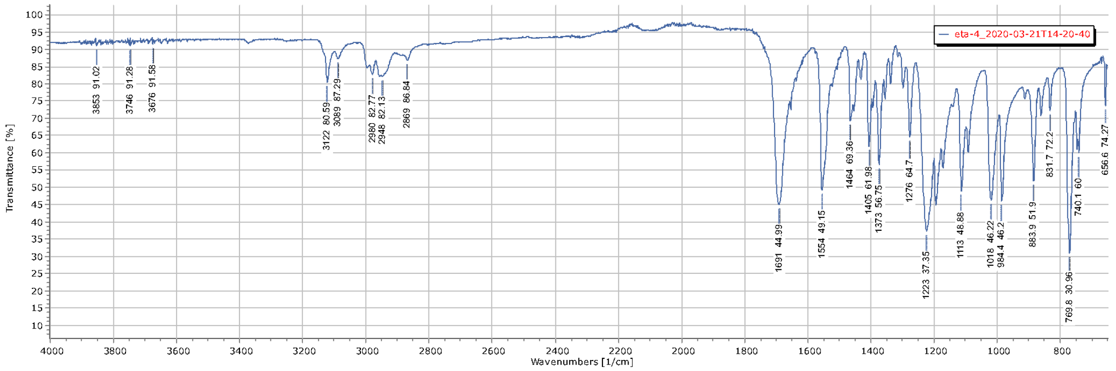

Compound 5f

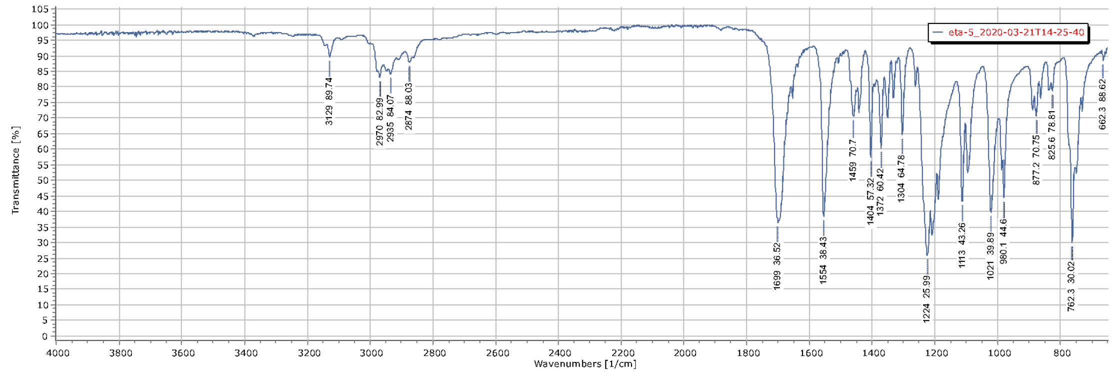

Compound 6f

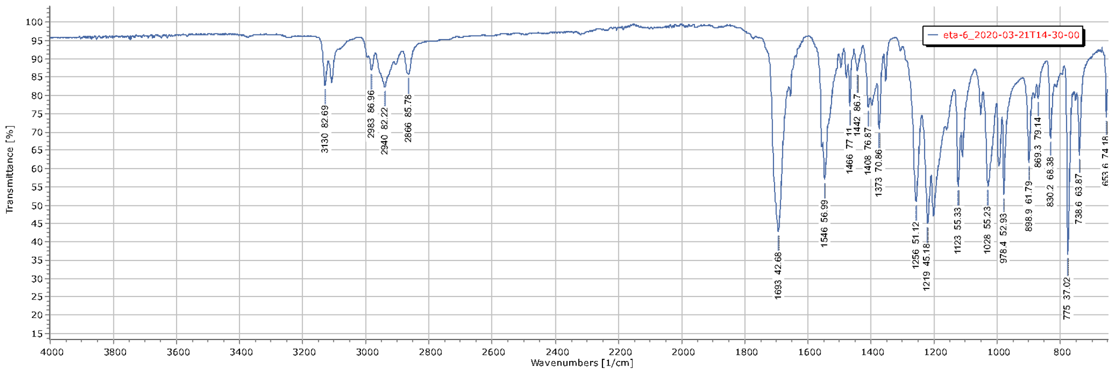

Compound 1g

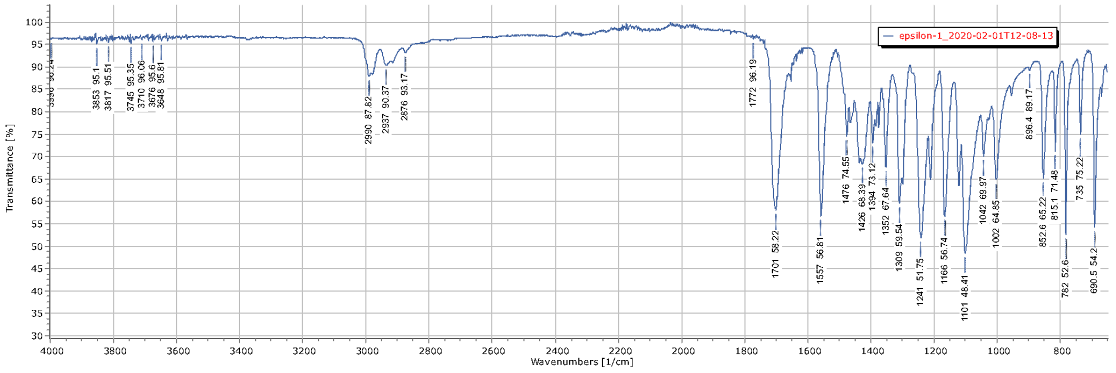

Compound 3g

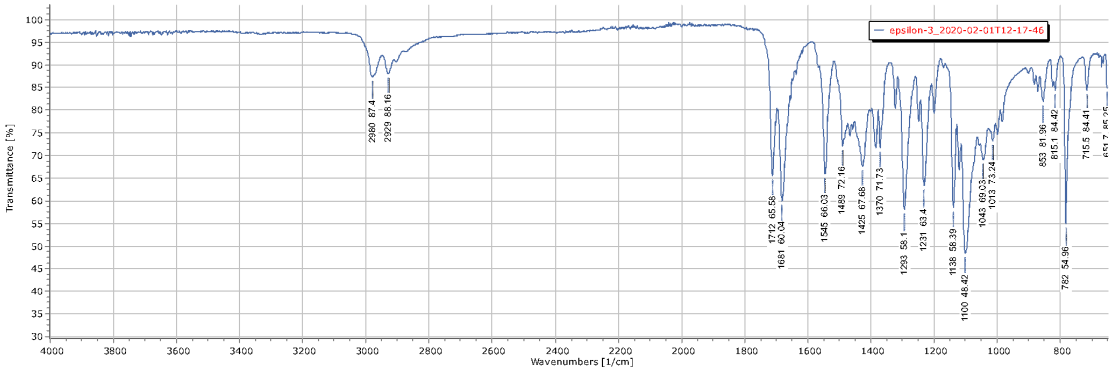

Compound 4g

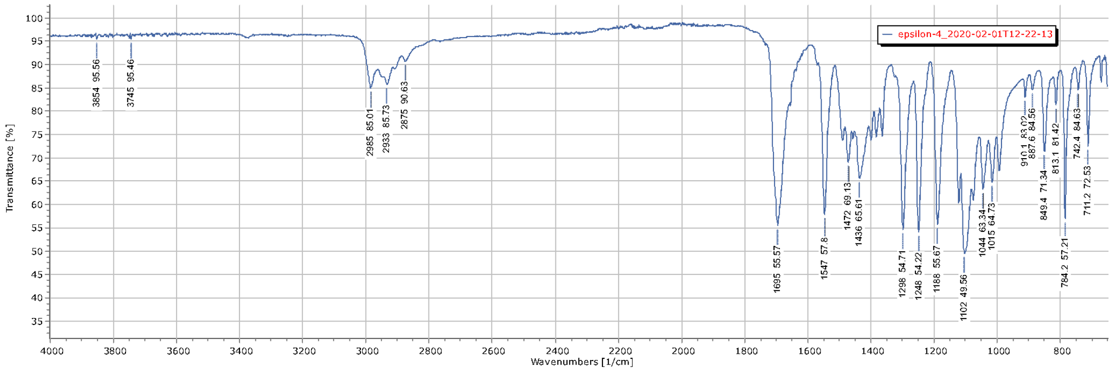

Compound 5g

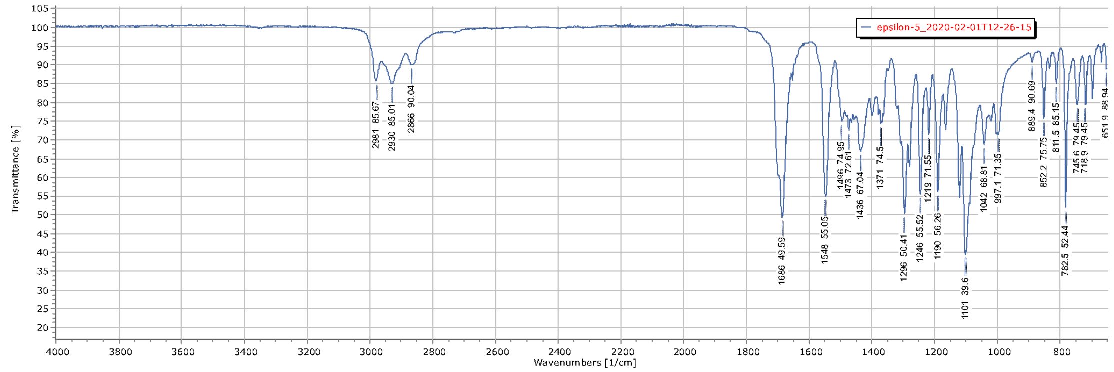

Compound 6g

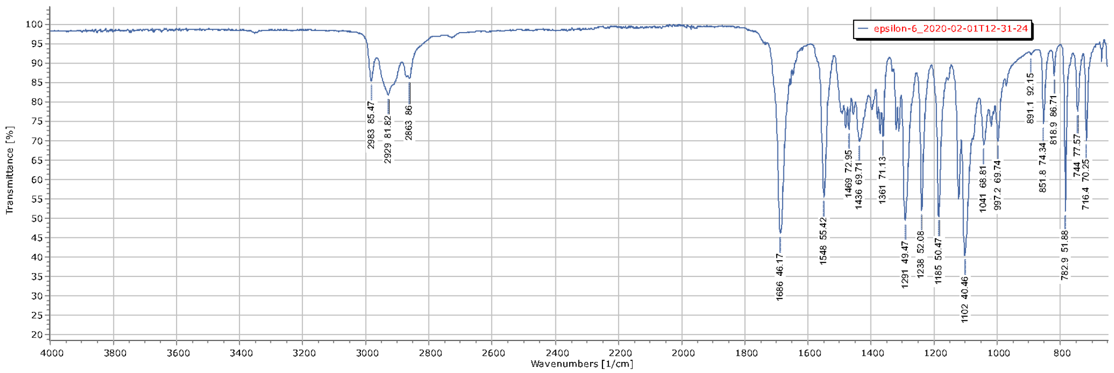

NMR ^{1}H and ^{13}C spectra of bis(pyrazol-1-yl)alkane derivatives

Compound 4b

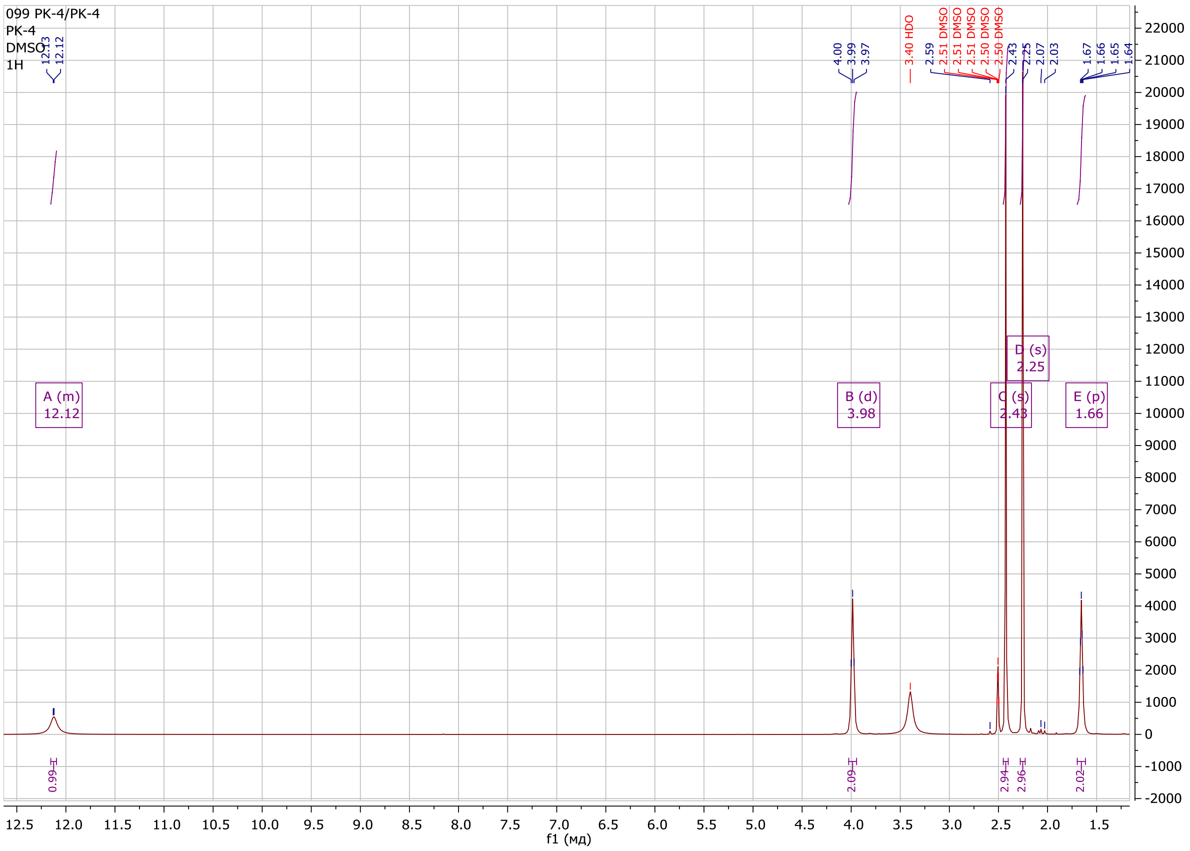

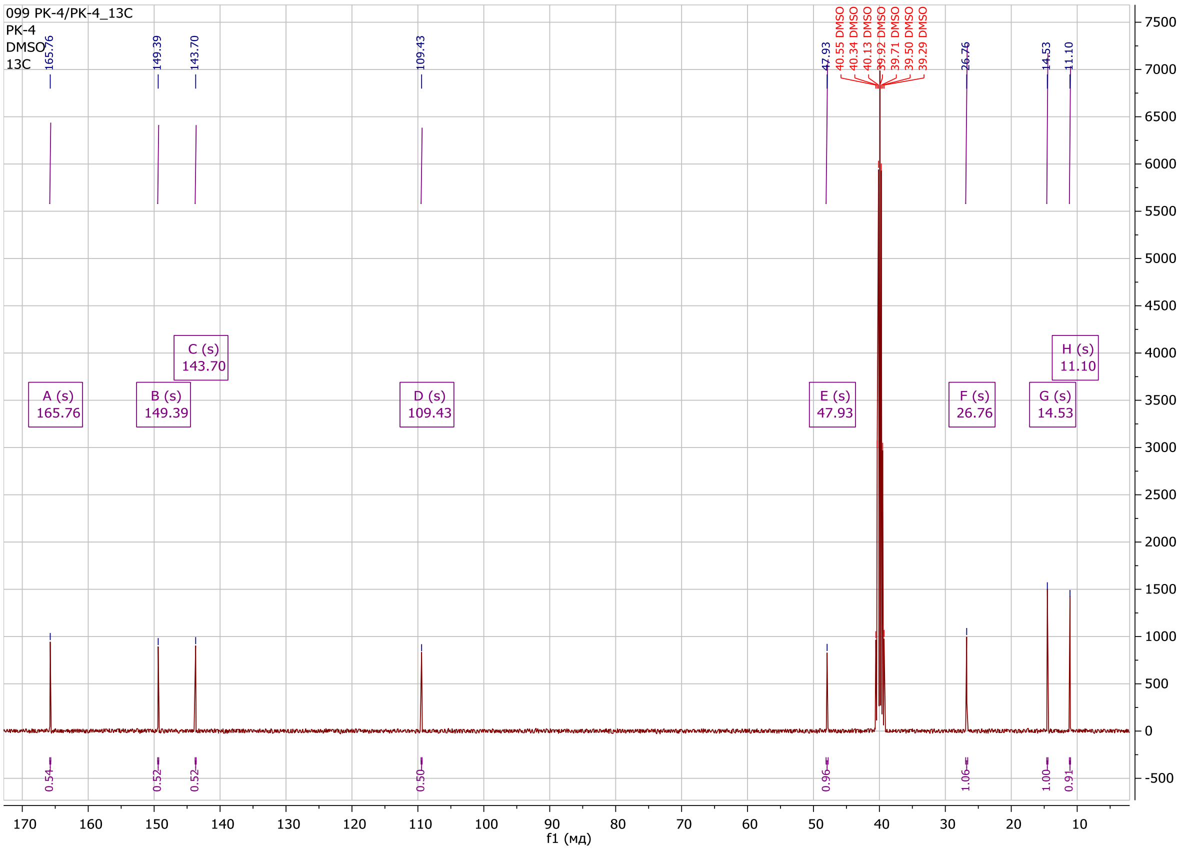

Compound 5b

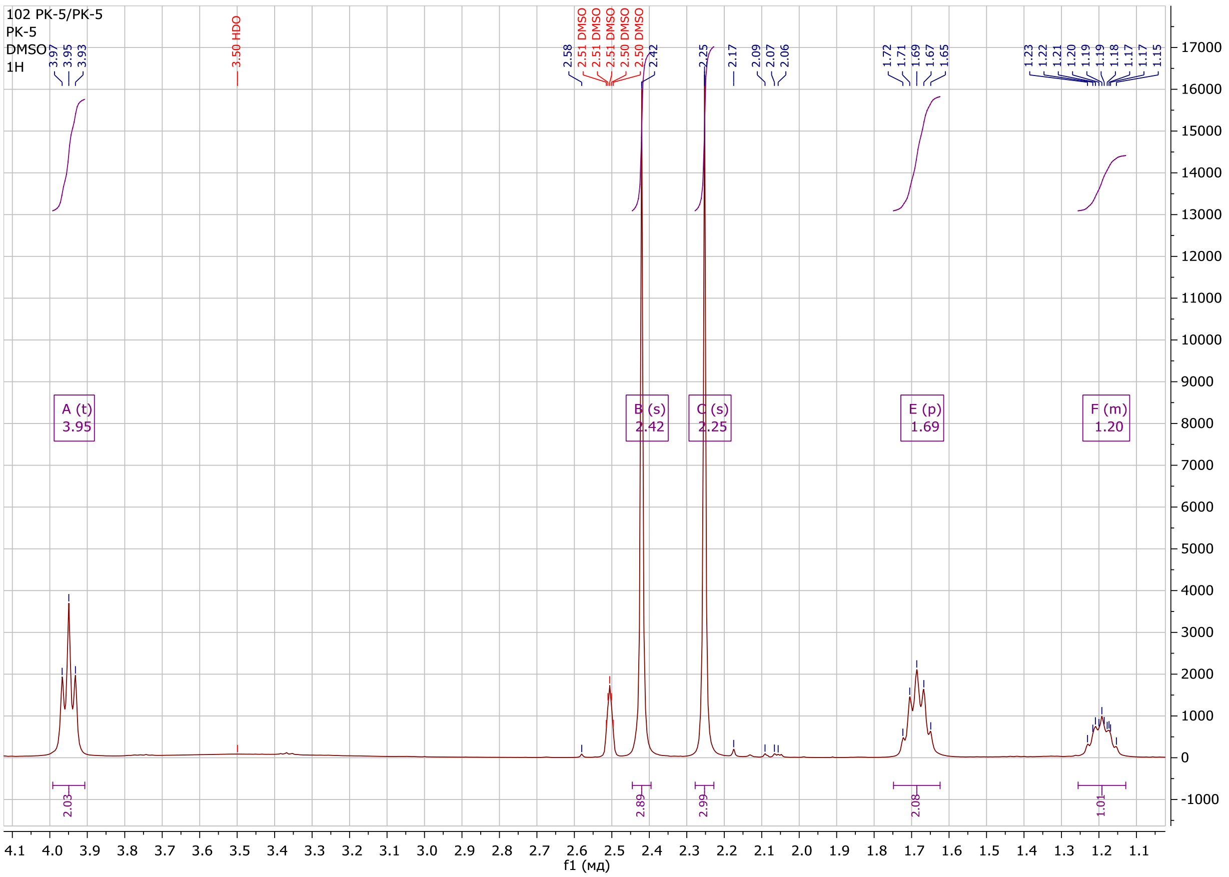
102 PK-5/PK-5
PK-5
DMSO
1H
3.50 HDO
A (t)
3.95
B (s)
2.42
C (s)
2.25
E (p)
1.69
F (m)
1.20
2.03
2.89
2.99
2.08
1.01
f1 (мд)

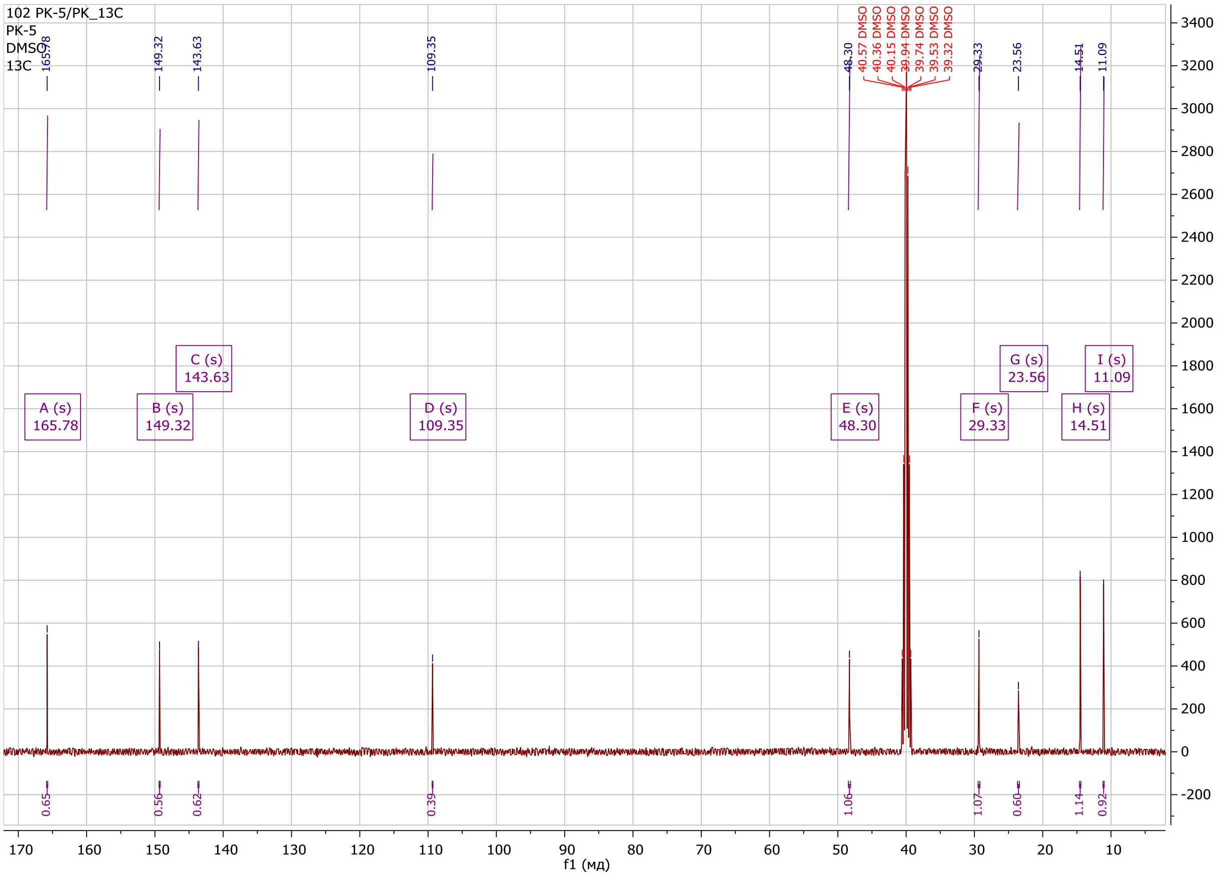
102 PK-5/PK_13C
PK-5
DMSO
13C
A (s)
165.78
B (s)
149.32
C (s)
143.63
D (s)
109.35
E (s)
48.30
F (s)
29.33
G (s)
23.56
H (s)
14.51
I (s)
11.09
f1 (мд)

Compound 6b

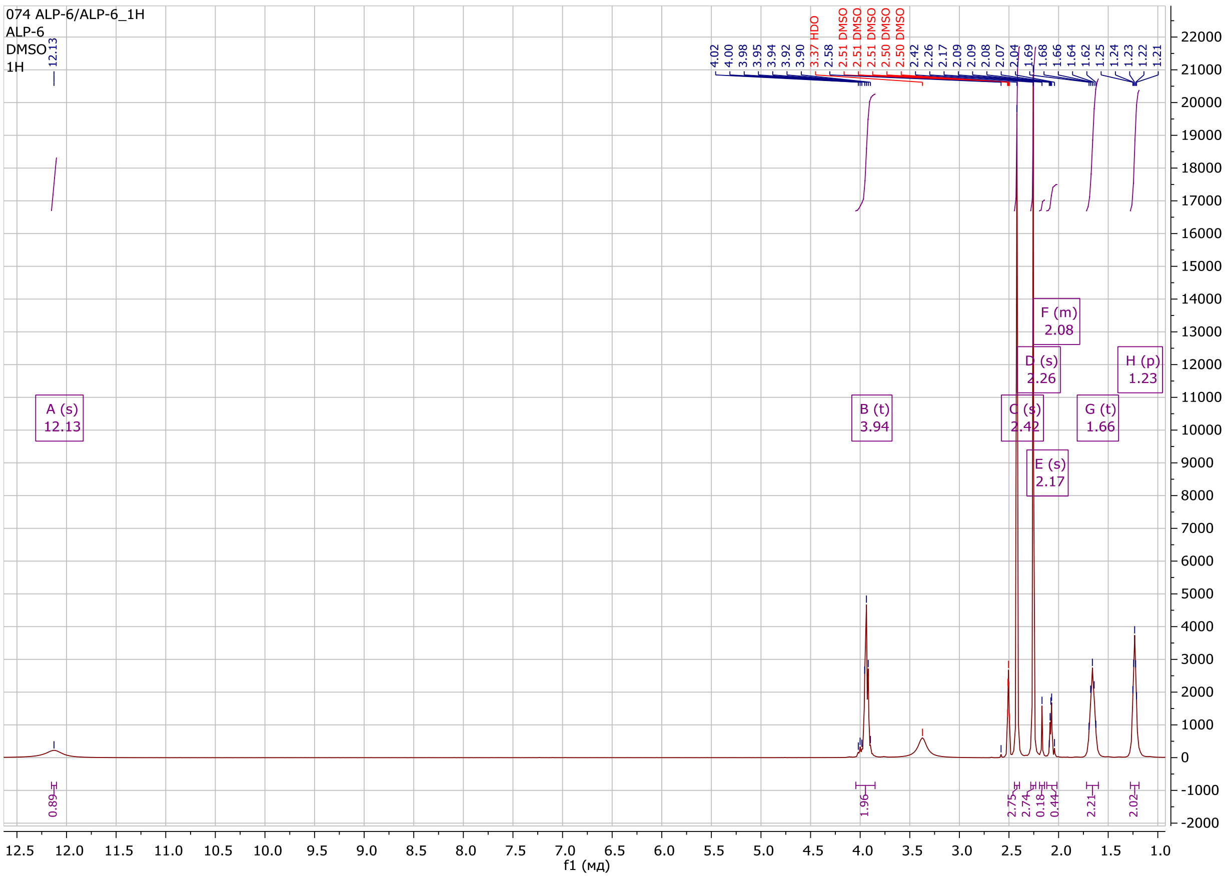
074 ALP-6/ALP-6_1H
ALP-6
DMSO
1H
A (s) 12.13
B (t) 3.94
C (s) 2.42
D (s) 2.26
E (s) 2.17
F (m) 2.08
G (t) 1.66
H (p) 1.23
f1 (мд)

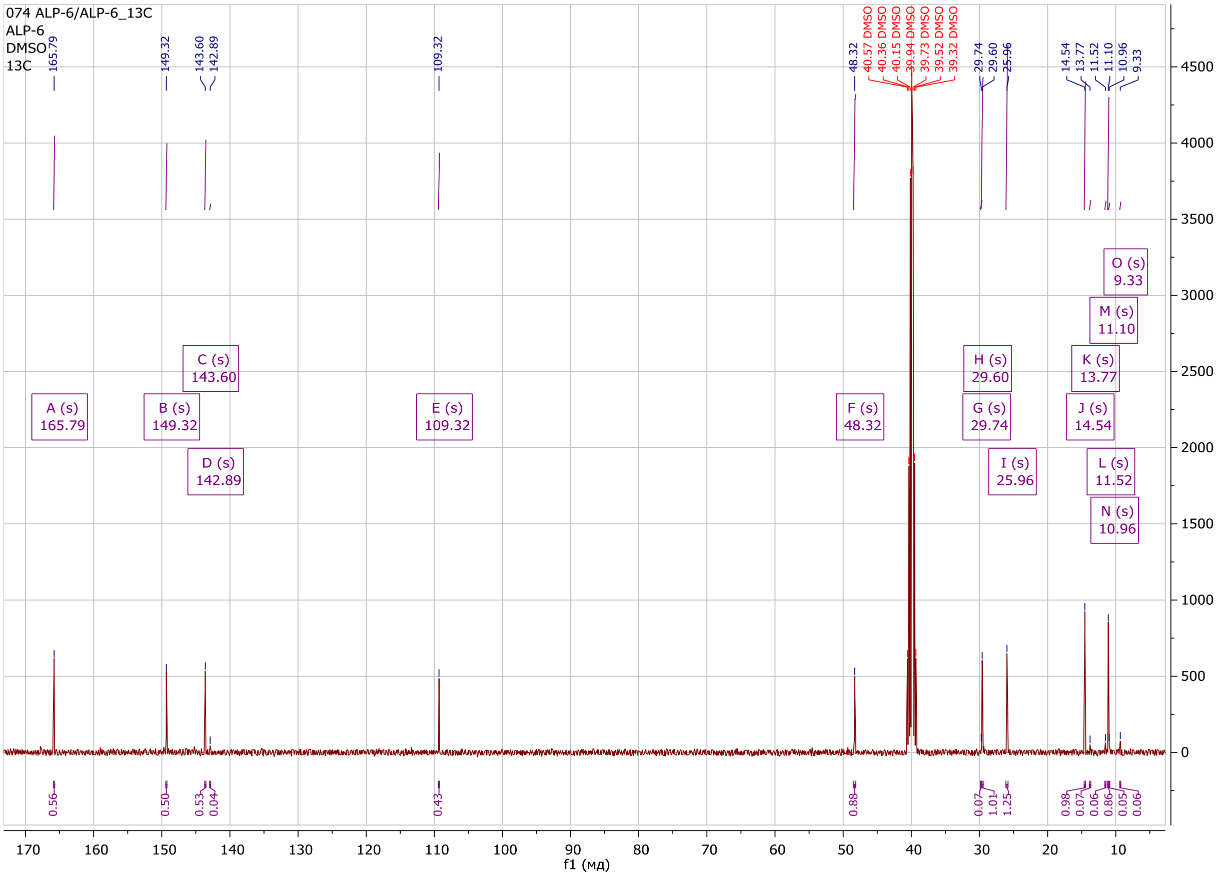
074 ALP-6/ALP-6_13C
ALP-6
DMSO
13C
A (s) 165.79
B (s) 149.32
C (s) 143.60
D (s) 142.89
E (s) 109.32
F (s) 48.32
G (s) 29.74
H (s) 29.60
I (s) 25.96
J (s) 14.54
K (s) 13.77
L (s) 11.52
M (s) 11.10
N (s) 10.96
O (s) 9.33
f1 (мд)

Compound 4c

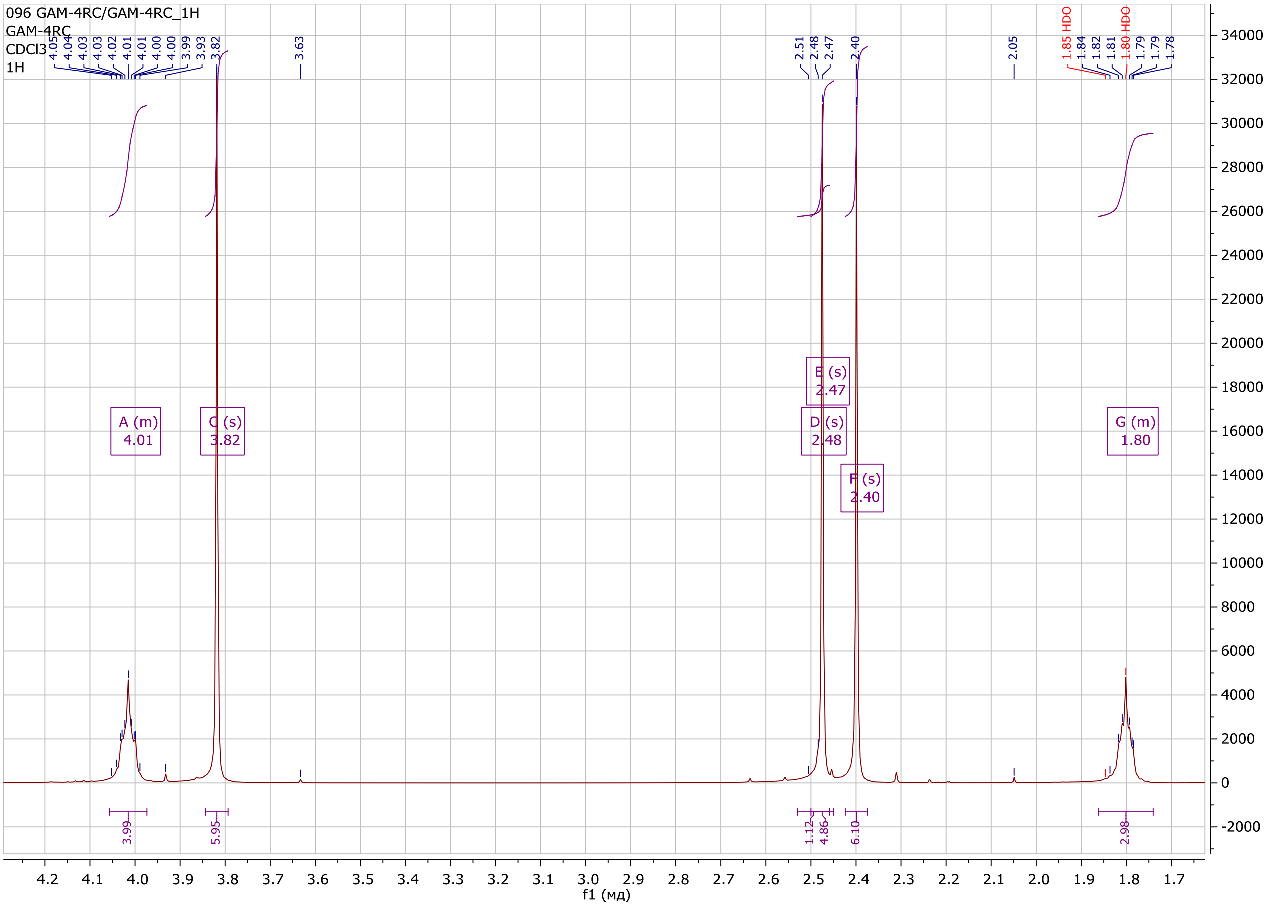

096 GAM-4RC/GAM-4RC_1H
GAM-4RC
CDCl3
1H
4.05 4.04 4.03 4.03 4.02 4.01 4.01 4.00 4.00 3.99 3.93 3.82
3.63
2.51 2.48 2.47
2.40
2.05
1.85 HDO 1.84 1.82 1.81 1.80 HDO 1.79 1.79 1.78
A (m) 4.01
C (s) 3.82
B (s) 2.47
D (s) 2.48
F (s) 2.40
G (m) 1.80
3.99
5.95
1.12
4.86
6.10
2.98
f1 (мд)

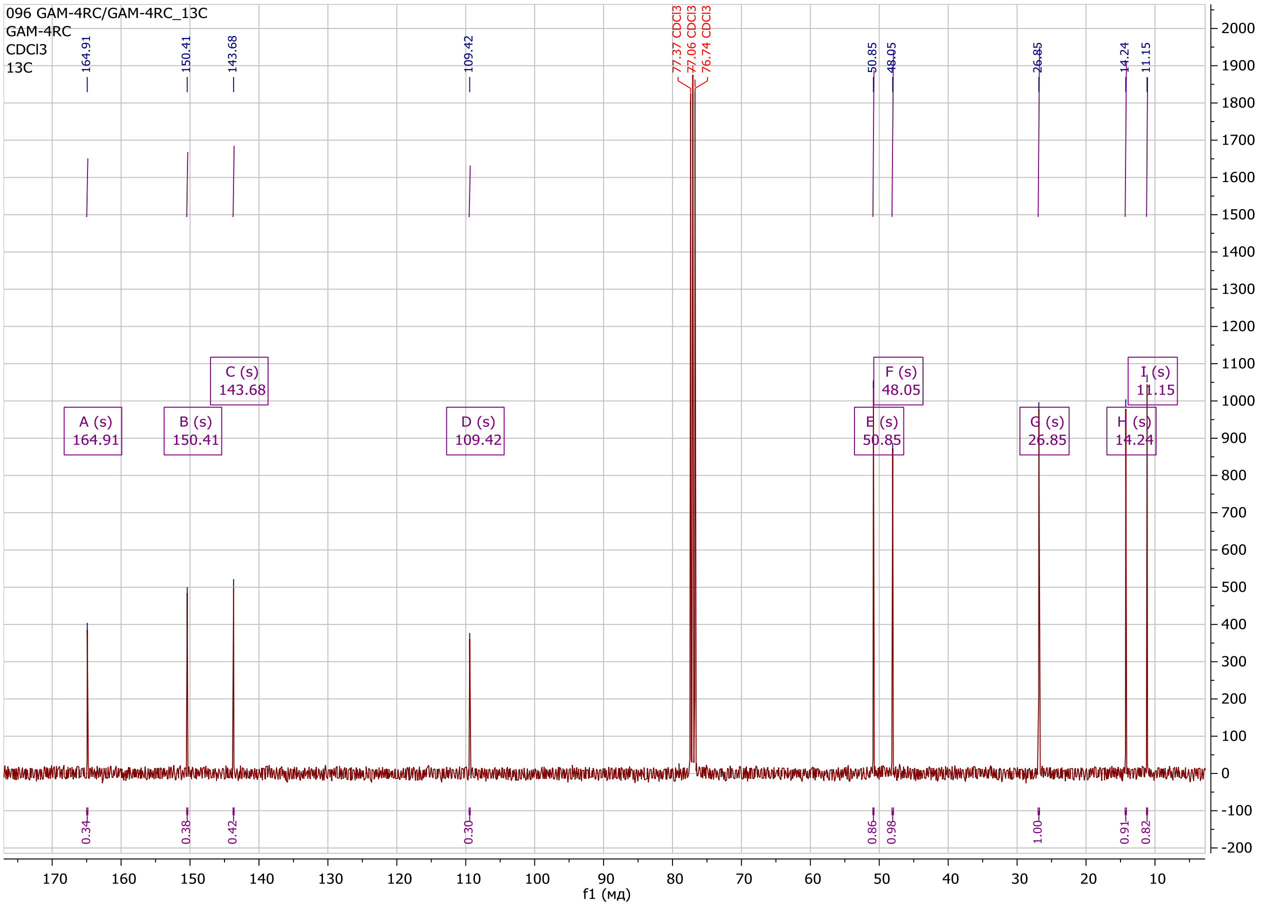

096 GAM-4RC/GAM-4RC_13C
GAM-4RC
CDCl3
13C
164.91
150.41
143.68
109.42
77.37 CDCl3 77.06 CDCl3 76.74 CDCl3
50.85
48.05
26.85
14.24
11.15
A (s) 164.91
B (s) 150.41
C (s) 143.68
D (s) 109.42
E (s) 50.85
F (s) 48.05
G (s) 26.85
H (s) 14.24
I (s) 11.15
0.34
0.38
0.42
0.30
0.86
0.98
1.00
0.91
0.82
f1 (мд)

Compound 5c

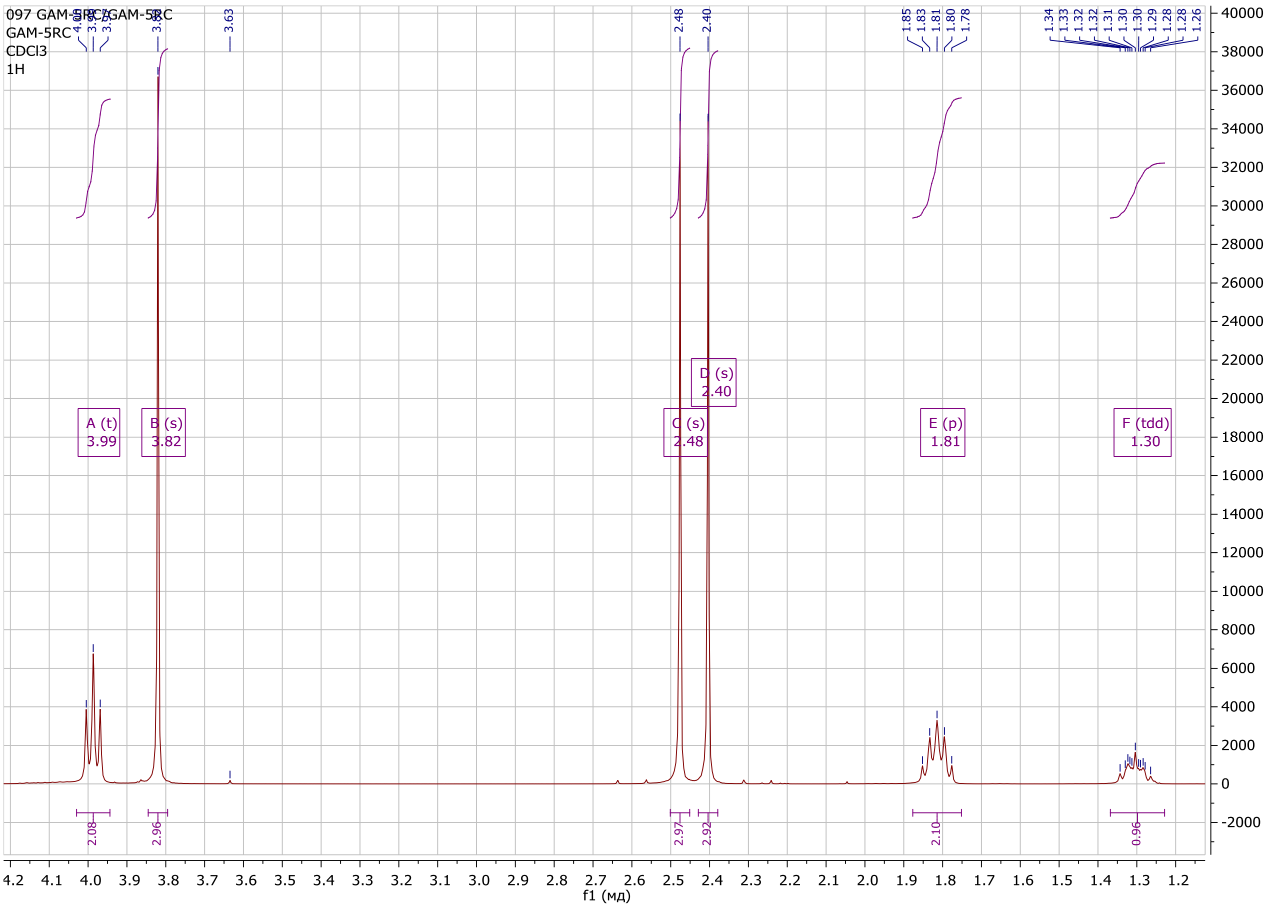

097 GAM-5RC/GAM-5RC
GAM-5RC
CDCl3
1H
A (t) 3.99
B (s) 3.82
C (s) 2.48
D (s) 2.40
E (p) 1.81
F (tdd) 1.30
2.08
2.96
2.97
2.92
2.10
0.96
f1 (мд)

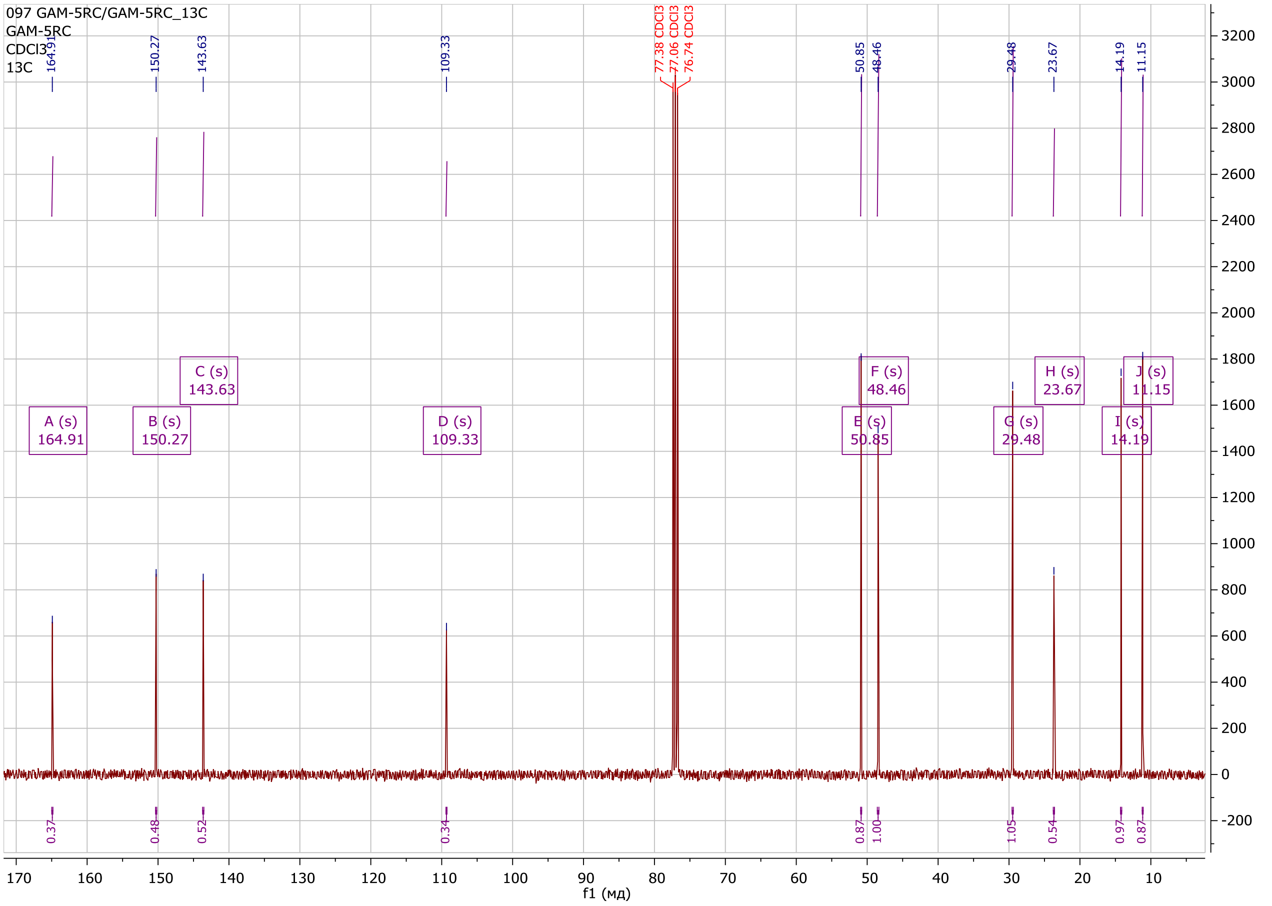

097 GAM-5RC/GAM-5RC_13C
GAM-5RC
CDCl3
13C
77.38 CDCl3
77.06 CDCl3
76.74 CDCl3
A (s) 164.91
B (s) 150.27
C (s) 143.63
D (s) 109.33
E (s) 50.85
F (s) 48.46
G (s) 29.48
H (s) 23.67
I (s) 14.19
J (s) 11.15
f1 (мд)

Compound 6c

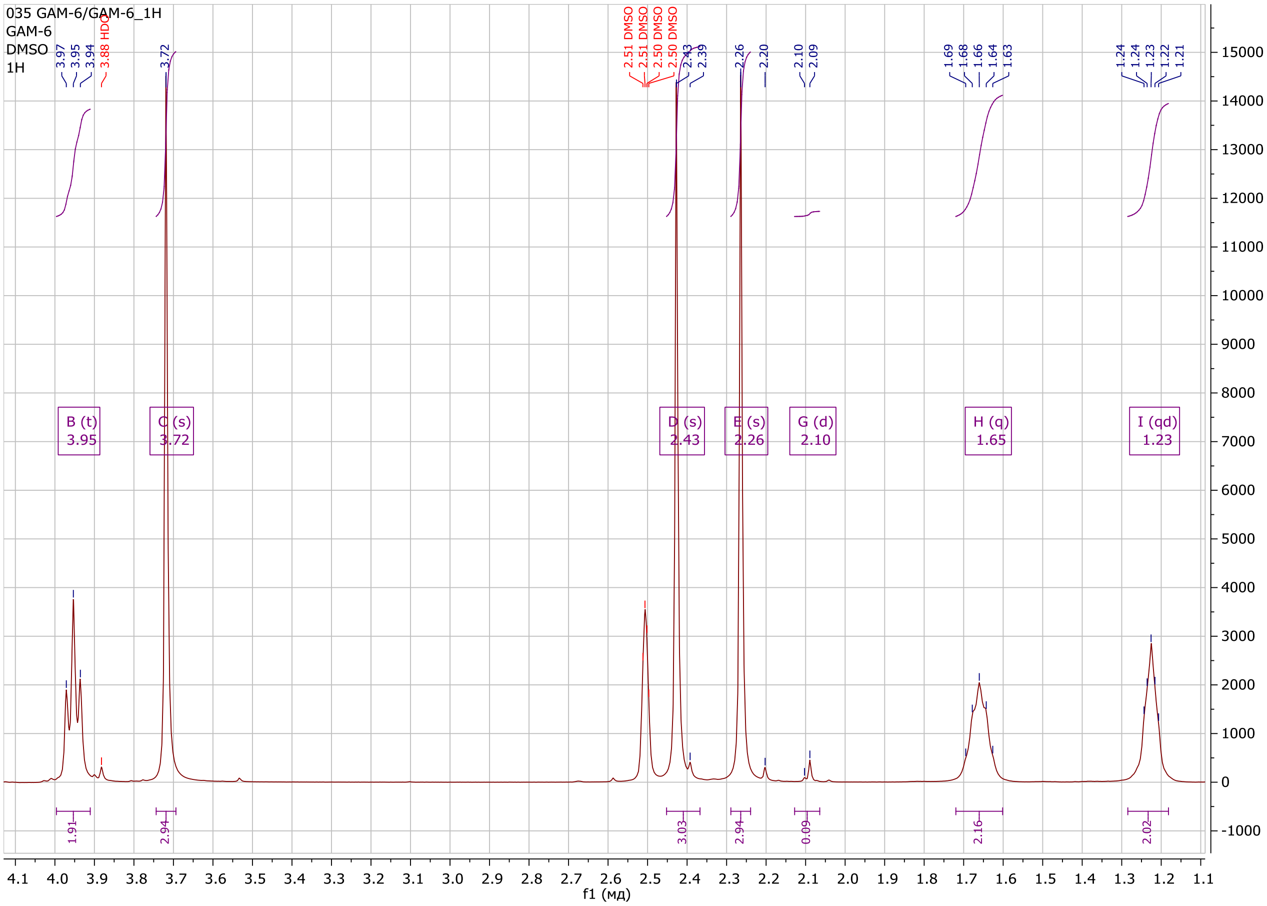
035 GAM-6/GAM-6_1H
GAM-6
DMSO
1H
B (t) 3.95
C (s) 3.72
D (s) 2.43
E (s) 2.26
G (d) 2.10
H (q) 1.65
I (qd) 1.23
f1 (мд)

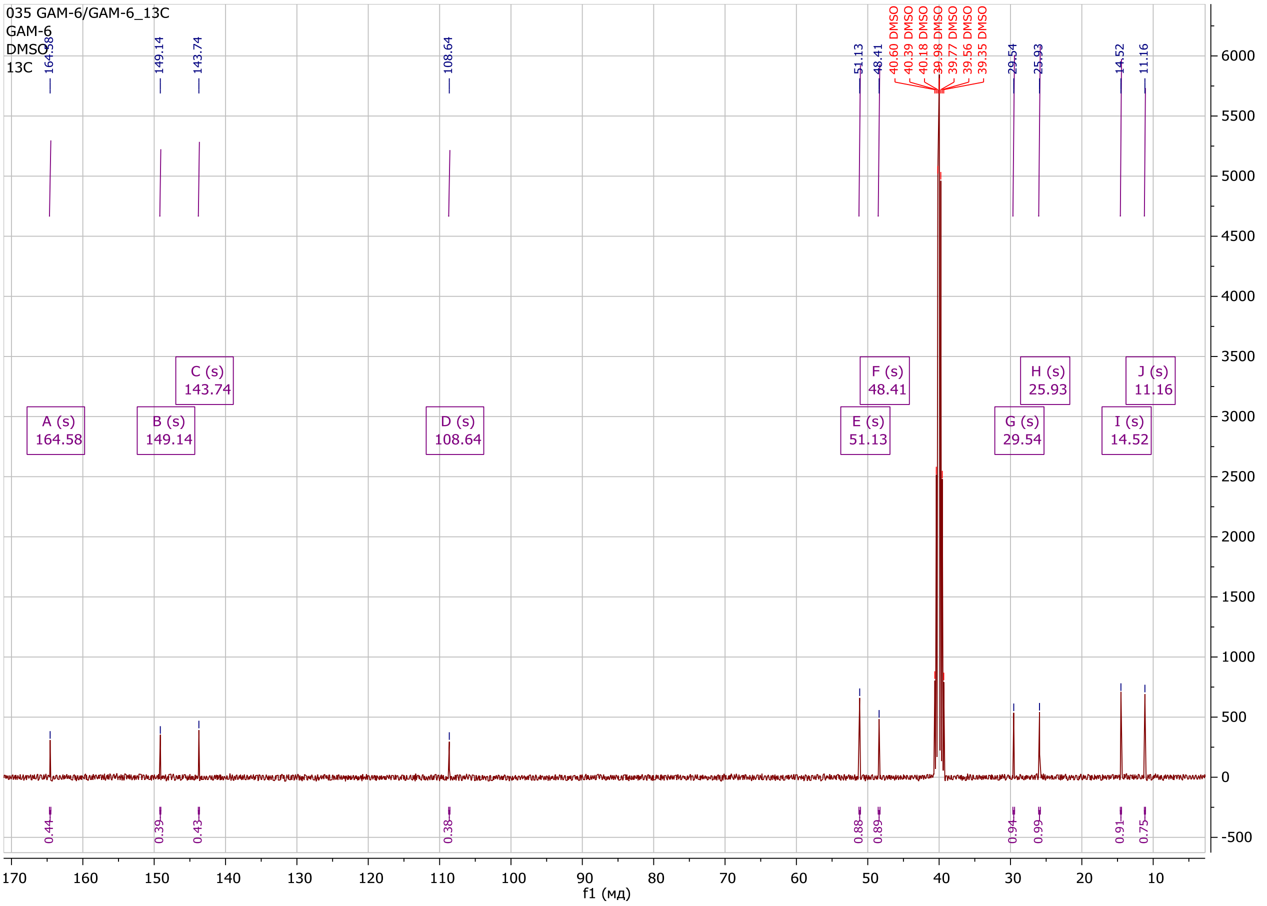
035 GAM-6/GAM-6_13C
GAM-6
DMSO
13C
A (s) 164.58
B (s) 149.14
C (s) 143.74
D (s) 108.64
E (s) 51.13
F (s) 48.41
G (s) 29.54
H (s) 25.93
I (s) 14.52
J (s) 11.16
f1 (мд)

Compound 1e

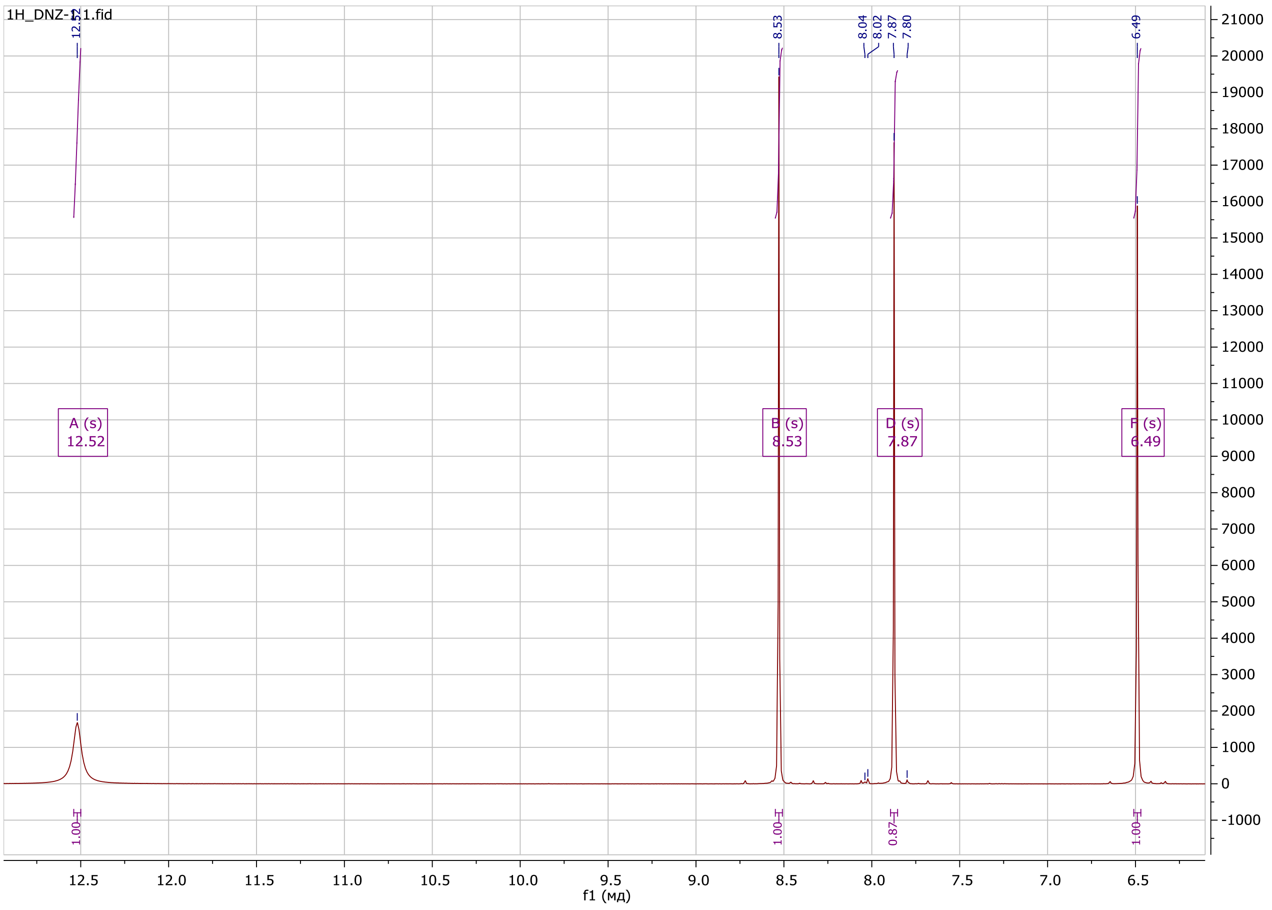
1H_DNZ-1.1.fid
12.52
8.53
8.04
8.02
7.87
7.80
6.49
A (s) 12.52
B (s) 8.53
D (s) 7.87
F (s) 6.49
1.00
1.00
0.87
1.00
f1 (мд)

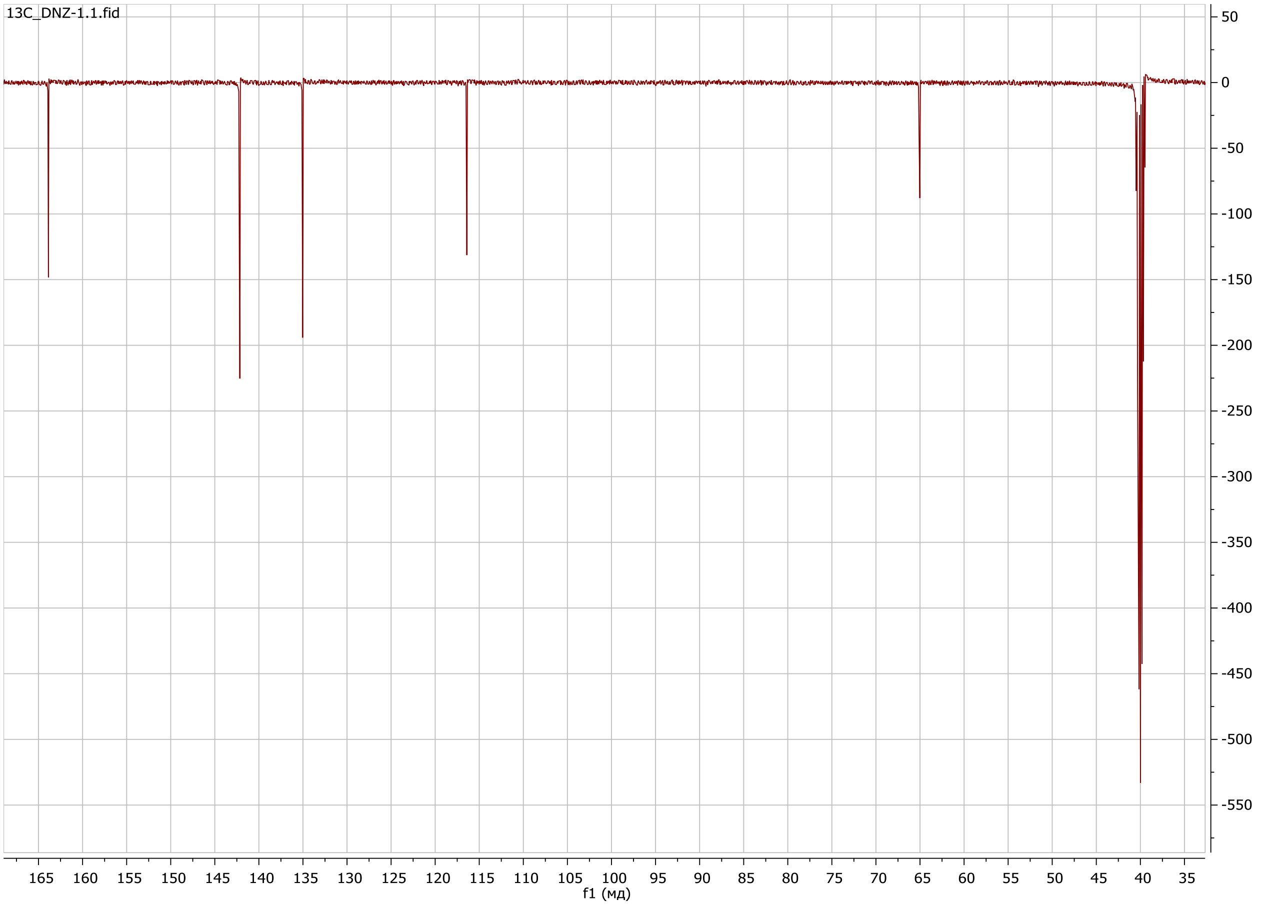
13C_DNZ-1.1.fid
f1 (мд)

Compound 4e

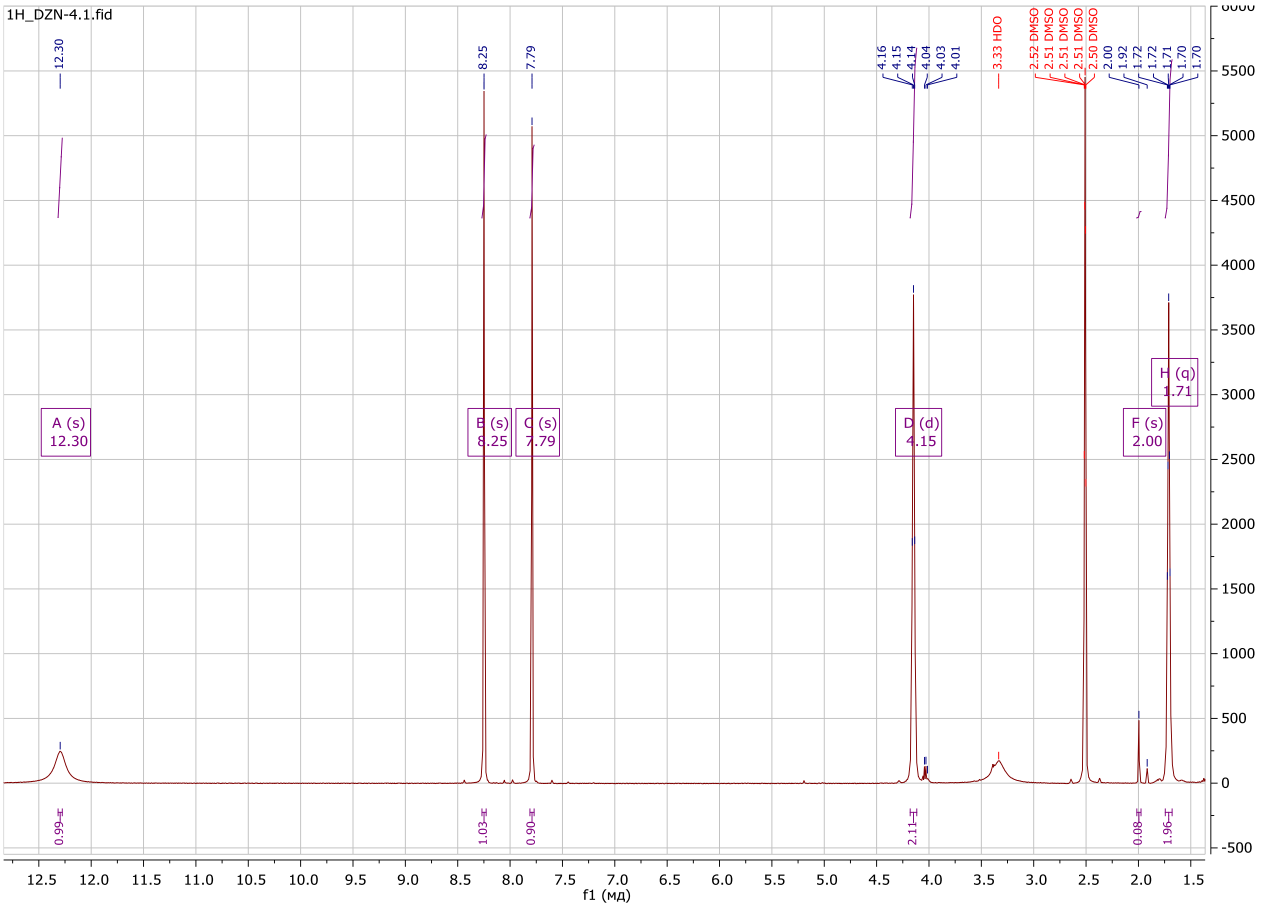

1H_DZN-4.1.fid
12.30
8.25
7.79
4.16
4.15
4.14
4.04
4.03
4.01
3.33 HDO
2.52 DMSO
2.51 DMSO
2.51 DMSO
2.51 DMSO
2.50 DMSO
2.00
1.92
1.72
1.72
1.71
1.70
1.70
A (s) 12.30
B (s) 8.25
C (s) 7.79
D (d) 4.15
F (s) 2.00
H (q) 1.71
0.99
1.03
0.90
2.11
0.08
1.96
f1 (мд)

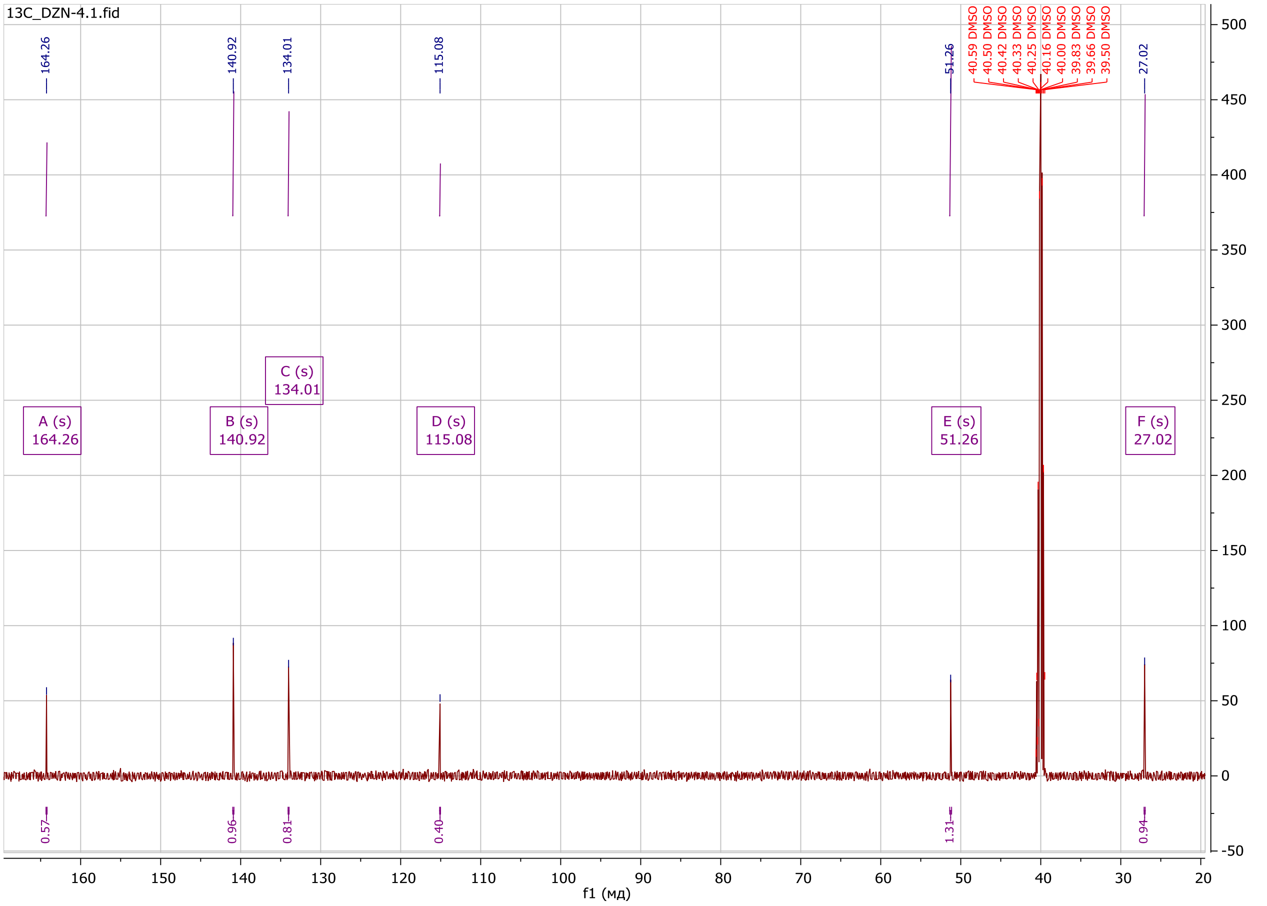

13C_DZN-4.1.fid
164.26
140.92
134.01
115.08
51.26
40.59 DMSO
40.50 DMSO
40.42 DMSO
40.33 DMSO
40.25 DMSO
40.16 DMSO
40.00 DMSO
39.83 DMSO
39.66 DMSO
39.50 DMSO
27.02
A (s) 164.26
B (s) 140.92
C (s) 134.01
D (s) 115.08
E (s) 51.26
F (s) 27.02
0.57
0.96
0.81
0.40
1.31
0.94
f1 (мд)

Compound 5e

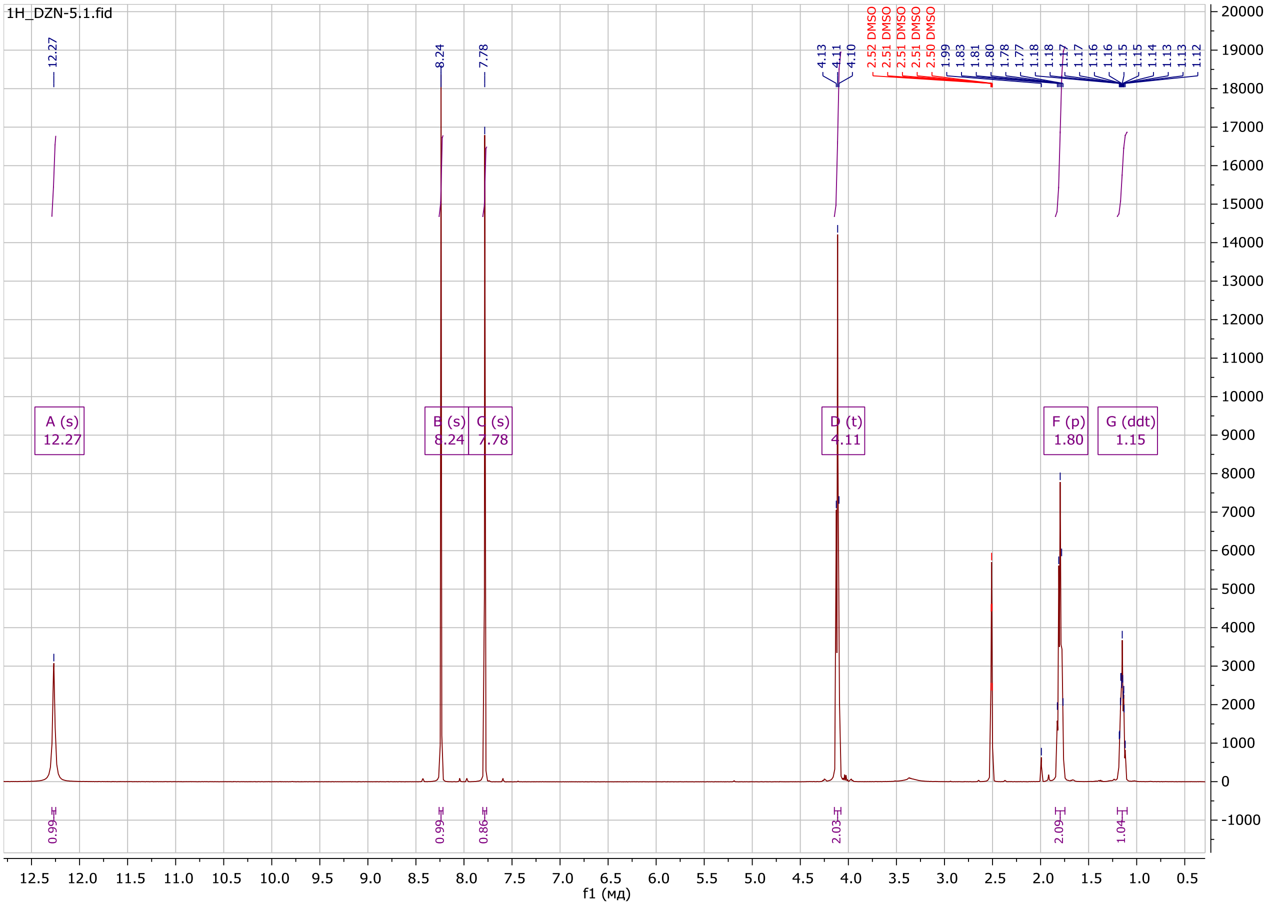
1H_DZN-5.1.fid
A (s) 12.27
B (s) 8.24
C (s) 7.78
D (t) 4.11
F (p) 1.80
G (ddt) 1.15
f1 (мд)

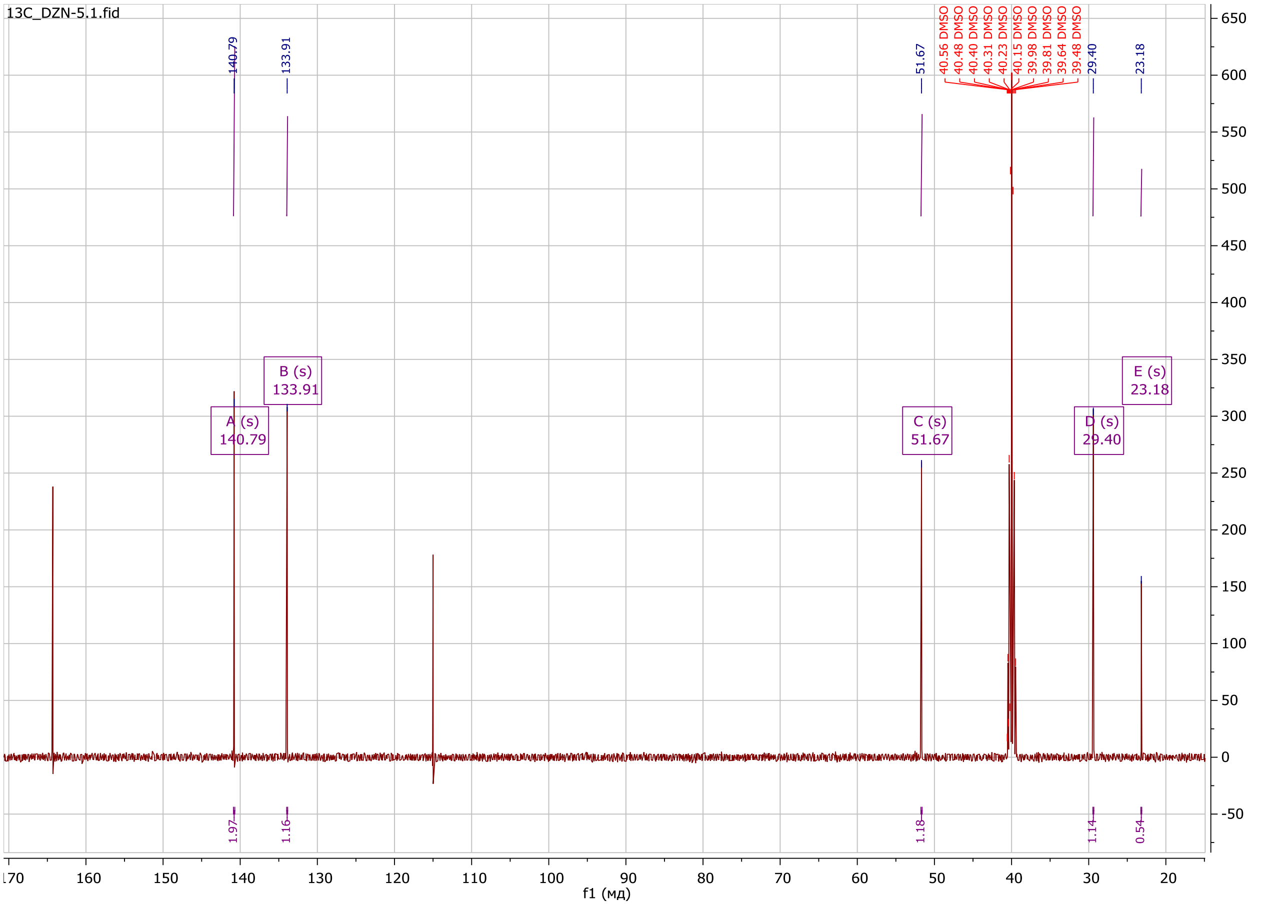
13C_DZN-5.1.fid
A (s) 140.79
B (s) 133.91
C (s) 51.67
D (s) 29.40
E (s) 23.18
f1 (мд)

Compound 6e

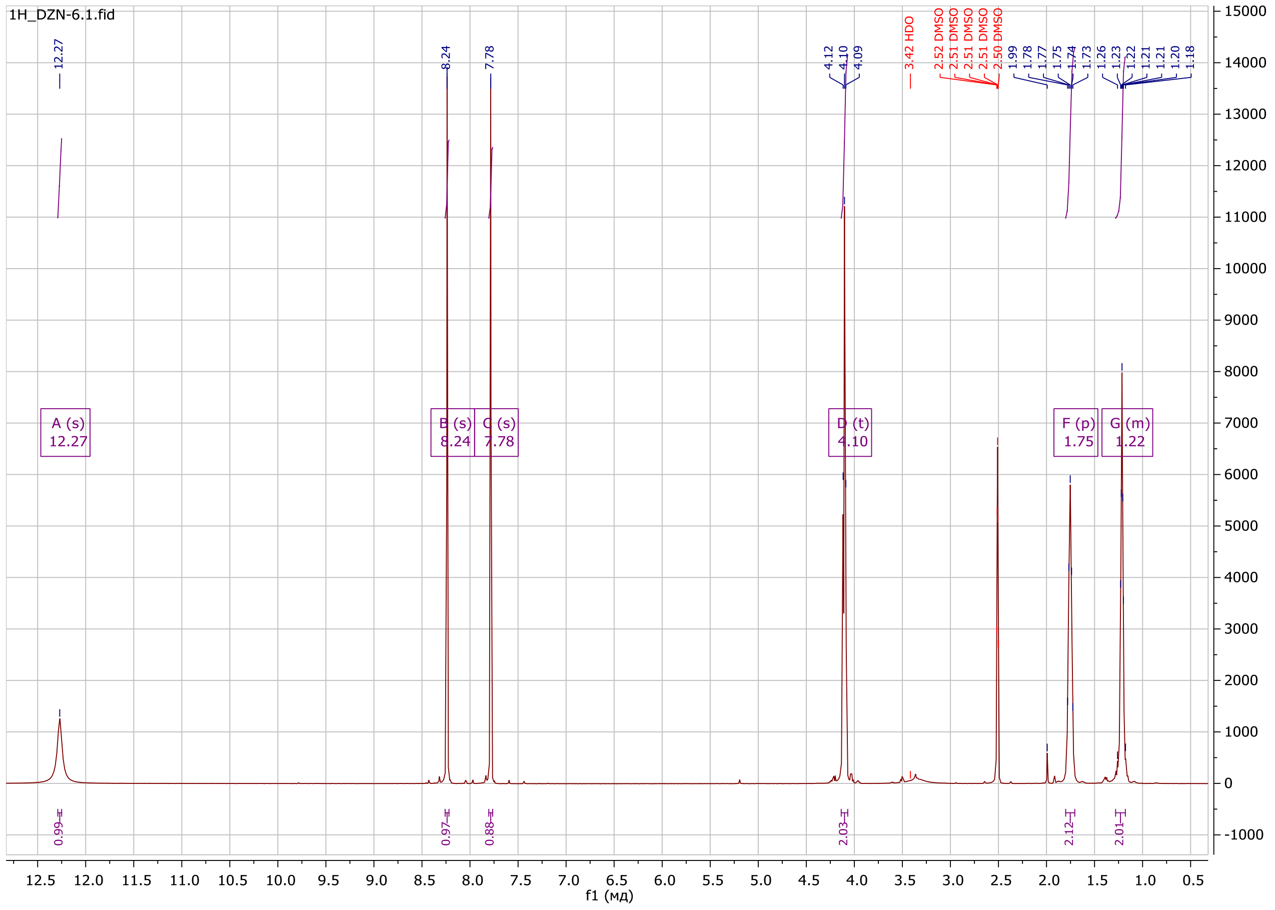
1H_DZN-6.1.fid
12.27
8.24
7.78
4.12
4.10
4.09
3.42 HDO
2.52 DMSO
2.51 DMSO
2.51 DMSO
2.51 DMSO
2.50 DMSO
1.99
1.78
1.77
1.75
1.74
1.73
1.26
1.23
1.22
1.21
1.20
1.18
A (s)
12.27
B (s)
8.24
C (s)
7.78
D (t)
4.10
F (p)
1.75
G (m)
1.22
0.99
0.97
0.88
2.03
2.12
2.01
f1 (мд)

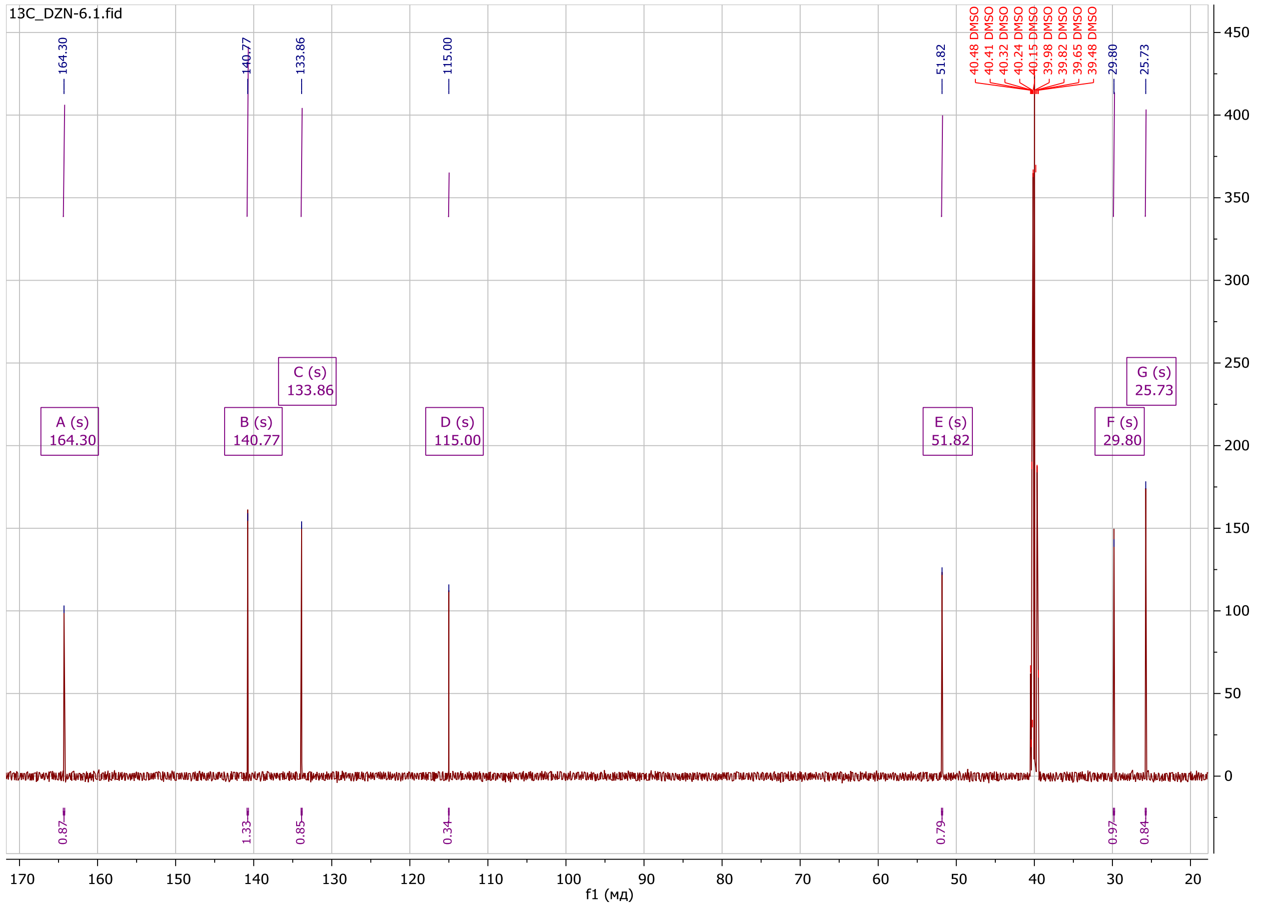
13C_DZN-6.1.fid
164.30
140.77
133.86
115.00
51.82
40.48 DMSO
40.41 DMSO
40.32 DMSO
40.24 DMSO
40.15 DMSO
39.98 DMSO
39.82 DMSO
39.65 DMSO
39.48 DMSO
29.80
25.73
A (s)
164.30
B (s)
140.77
C (s)
133.86
D (s)
115.00
E (s)
51.82
F (s)
29.80
G (s)
25.73
0.87
1.33
0.85
0.34
0.79
0.97
0.84
f1 (мд)

Compound 1f

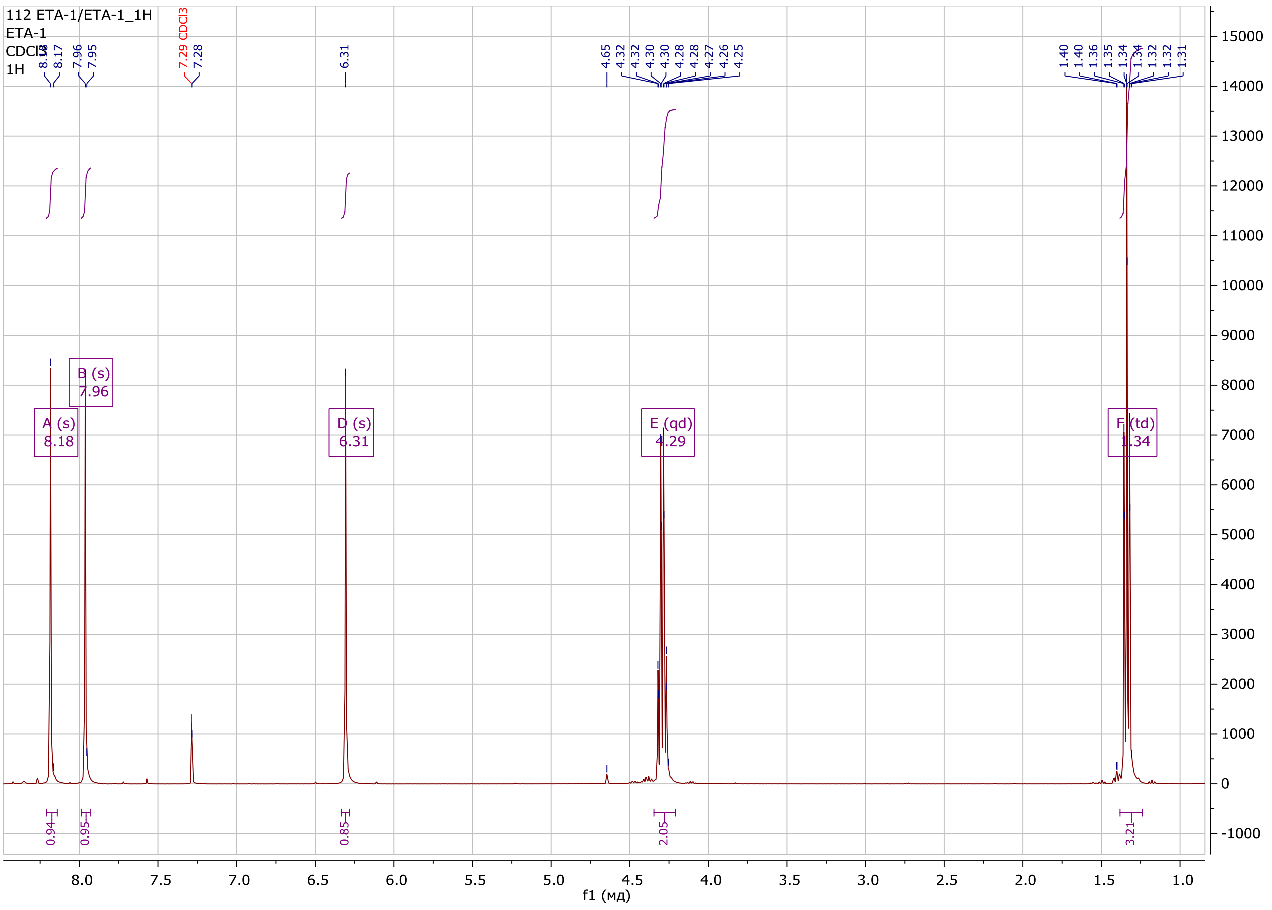

112 ETA-1/ETA-1_1H
ETA-1
CDCl3
1H
A (s) 8.18
B (s) 7.96
D (s) 6.31
E (qd) 4.29
F (td) 1.34
7.29 CDCl3
f1 (мд)

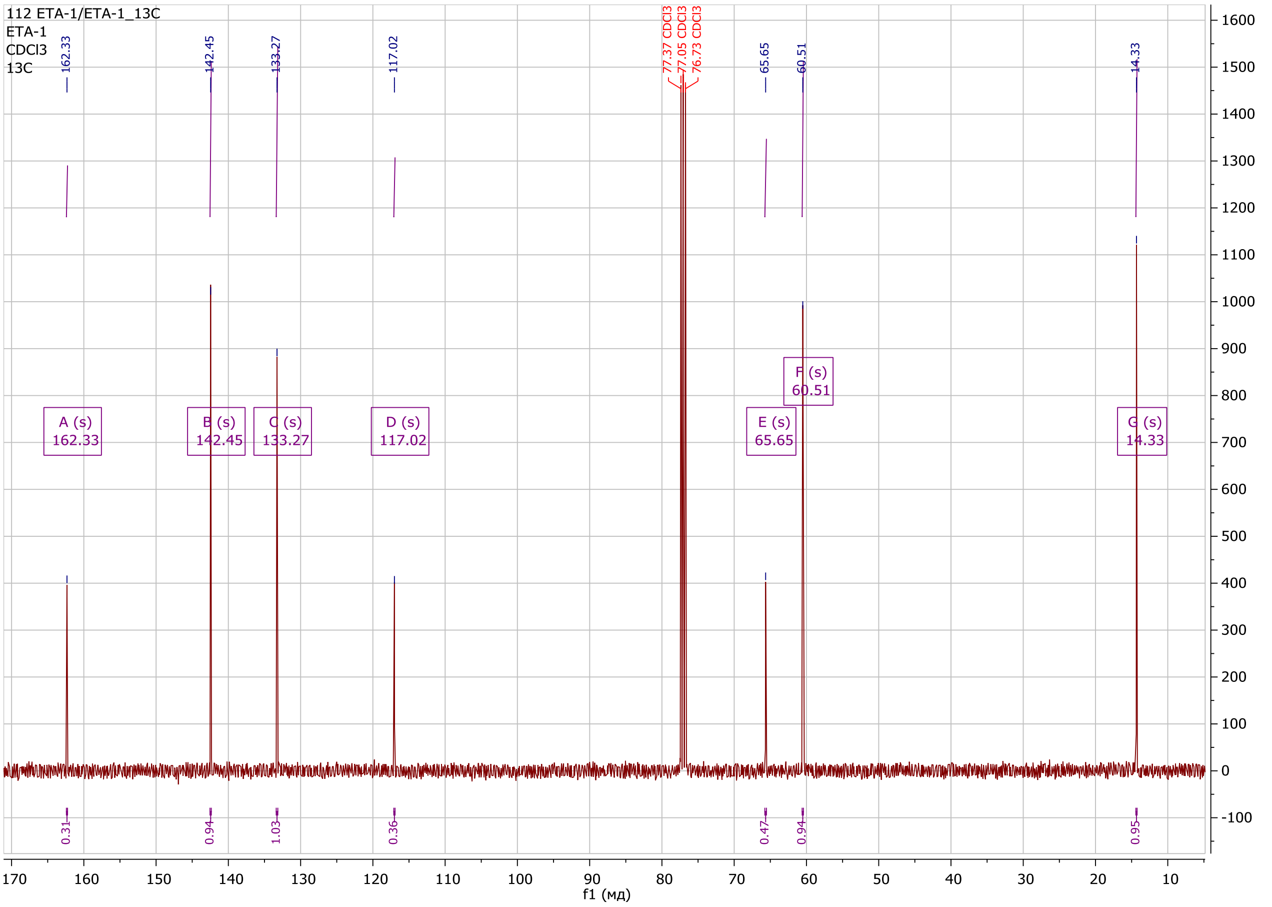

112 ETA-1/ETA-1_13C
ETA-1
CDCl3
13C
A (s) 162.33
B (s) 142.45
C (s) 133.27
D (s) 117.02
E (s) 65.65
F (s) 60.51
G (s) 14.33
77.37 CDCl3
77.05 CDCl3
76.73 CDCl3
f1 (мд)

Compound 3f

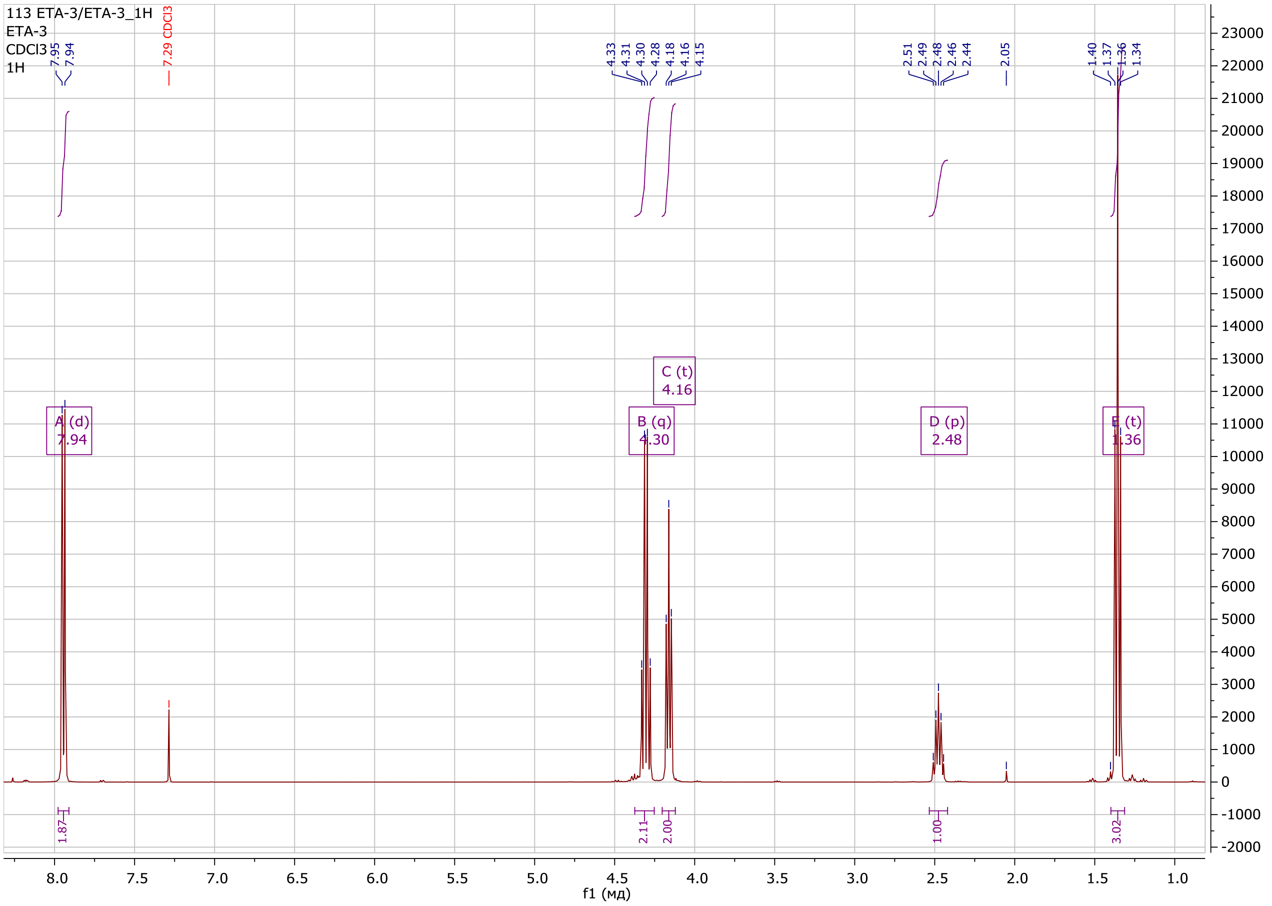

113 ETA-3/ETA-3_1H
ETA-3
CDCl3
1H
A (d) 7.94
B (q) 4.30
C (t) 4.16
D (p) 2.48
E (t) 1.36
f1 (мд)

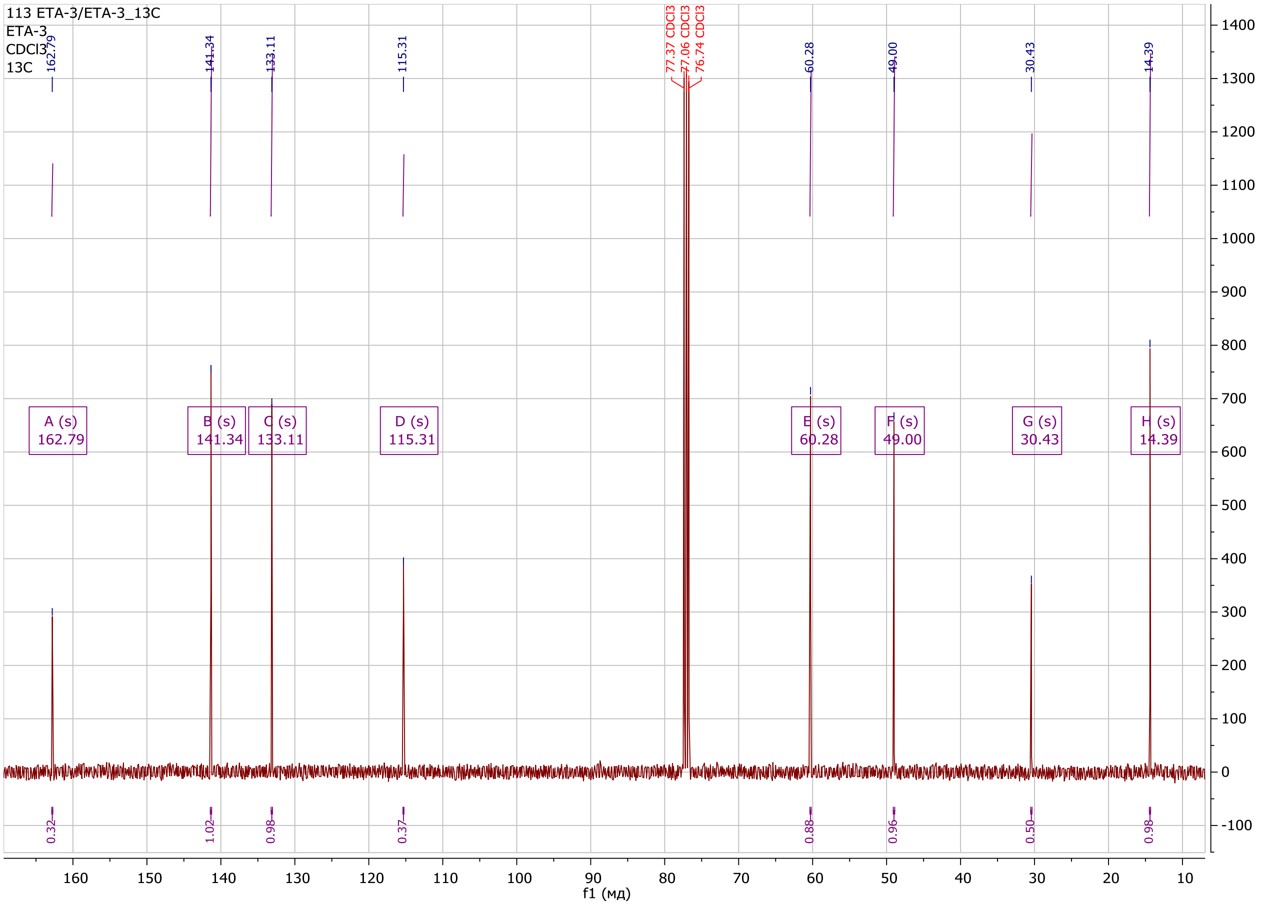

113 ETA-3/ETA-3_13C
ETA-3
CDCl3
13C
A (s) 162.79
B (s) 141.34
C (s) 133.11
D (s) 115.31
E (s) 60.28
F (s) 49.00
G (s) 30.43
H (s) 14.39
f1 (мд)

Compound 4f

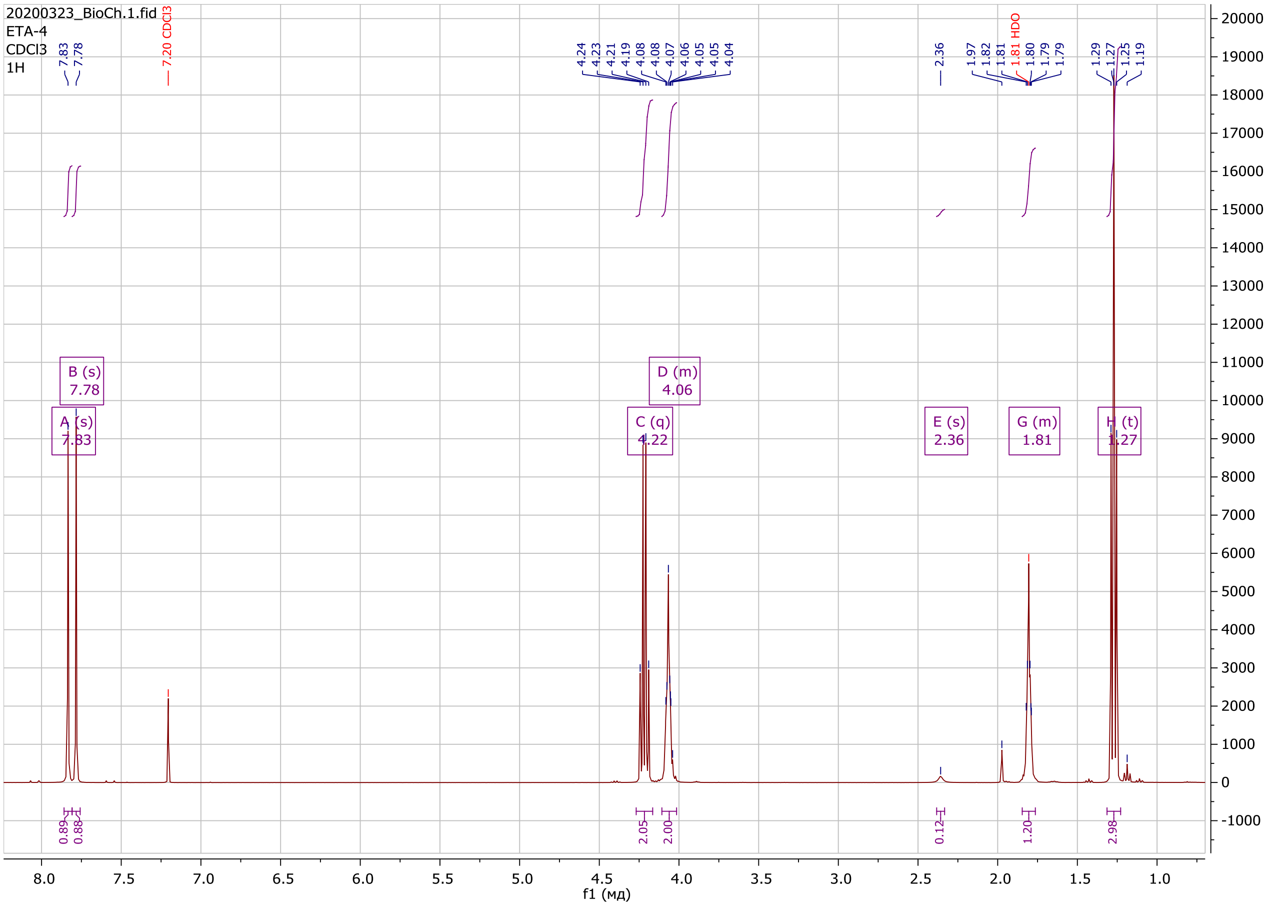
20200323_BioCh.1.fid
ETA-4
CDCl3
1H
A (s) 7.83
B (s) 7.78
C (q) 4.22
D (m) 4.06
E (s) 2.36
G (m) 1.81
H (t) 1.27
7.20 CDCl3
1.81 HDO
f1 (мд)

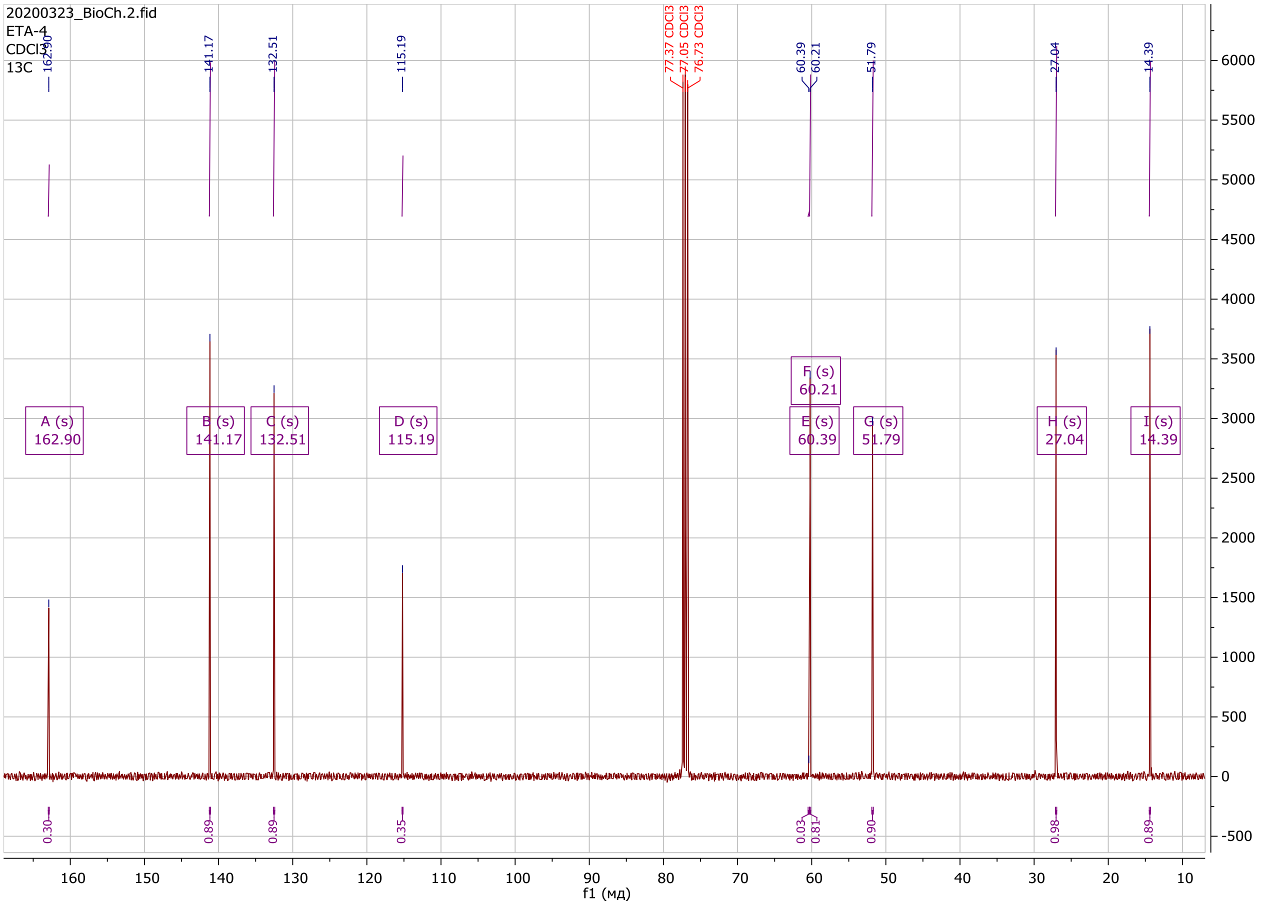
20200323_BioCh.2.fid
ETA-4
CDCl3
13C
A (s) 162.90
B (s) 141.17
C (s) 132.51
D (s) 115.19
E (s) 60.39
F (s) 60.21
G (s) 51.79
H (s) 27.04
I (s) 14.39
77.37 CDCl3
77.05 CDCl3
76.73 CDCl3
f1 (мд)

Compound 5f

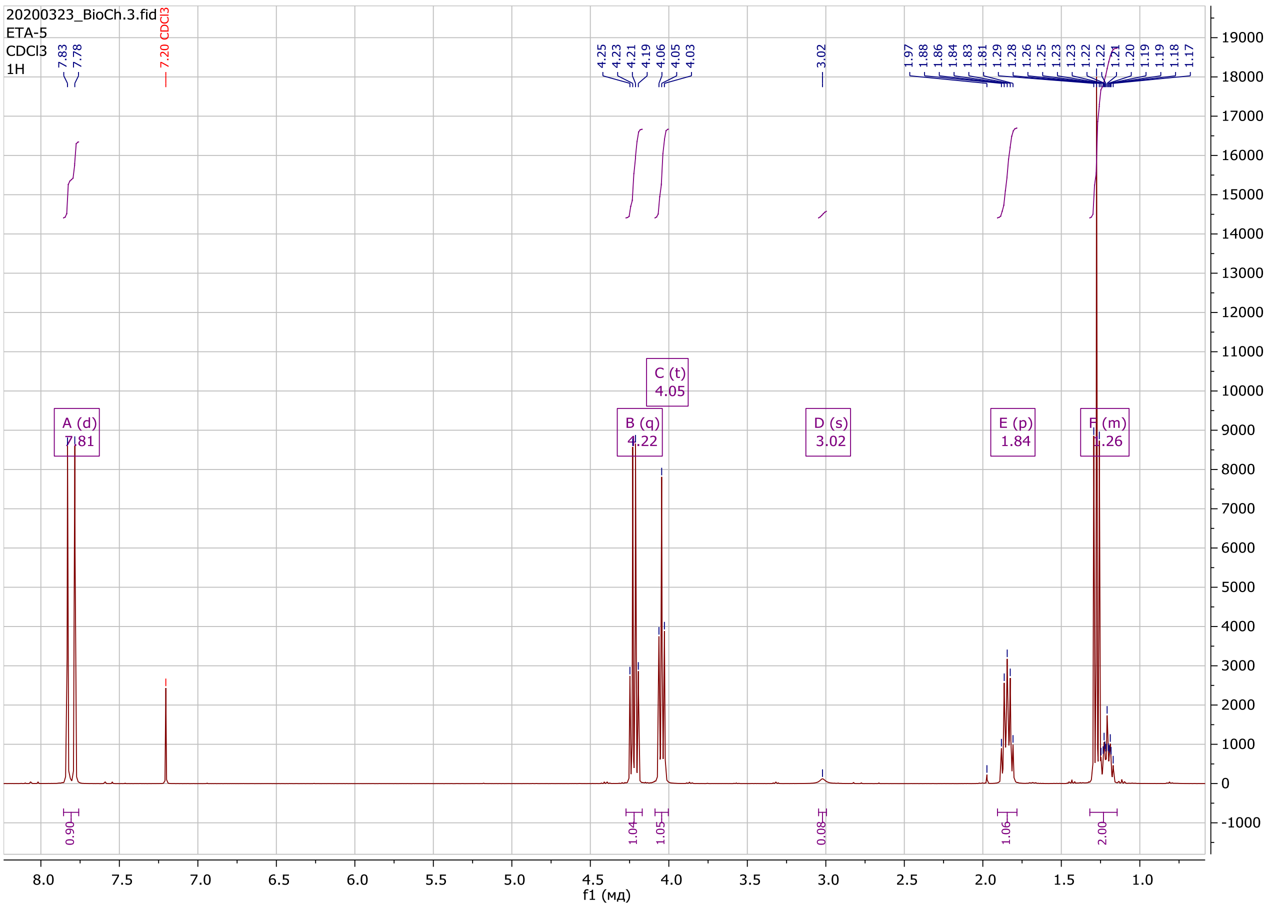
20200323_BioCh.3.fid
ETA-5
CDCl3
1H
A (d) 7.81
B (q) 4.22
C (t) 4.05
D (s) 3.02
E (p) 1.84
F (m) 1.26
f1 (мд)

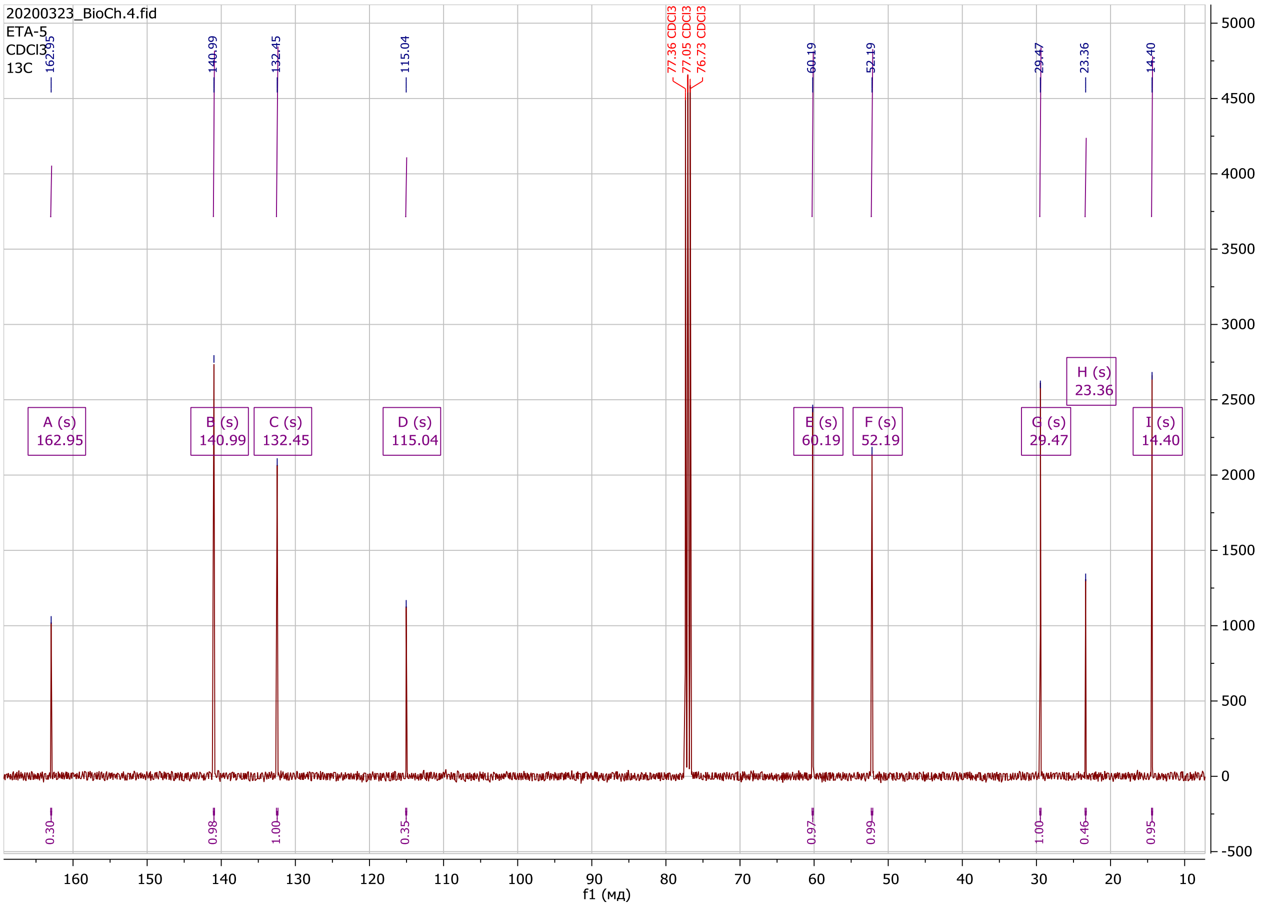
20200323_BioCh.4.fid
ETA-5
CDCl3
13C
A (s) 162.95
B (s) 140.99
C (s) 132.45
D (s) 115.04
E (s) 60.19
F (s) 52.19
G (s) 29.47
H (s) 23.36
I (s) 14.40
77.36 CDCl3
77.05 CDCl3
76.73 CDCl3
f1 (мд)

Compound 6f

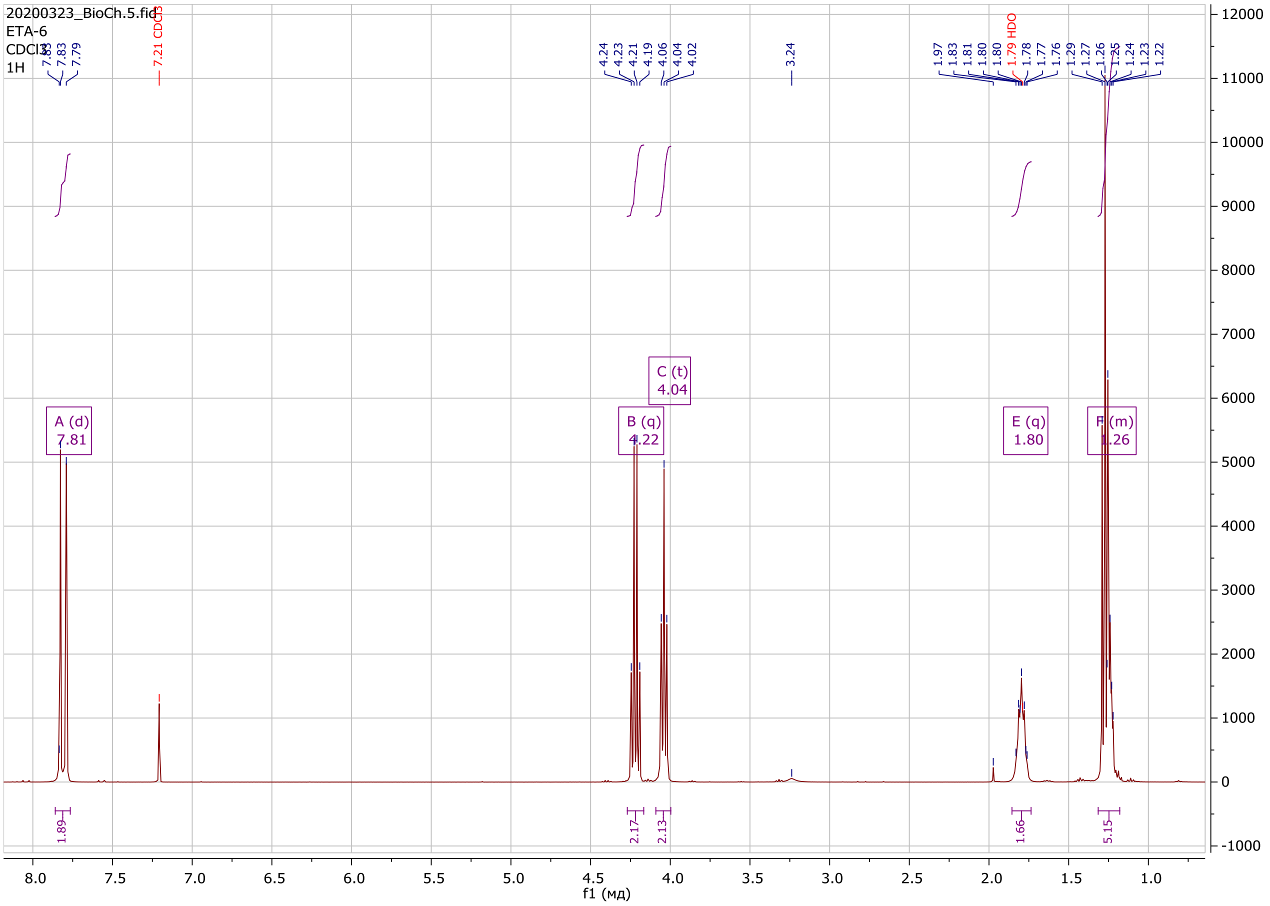

20200323_BioCh.5.fid
ETA-6
CDCl3
1H
7.21 CDCl3
1.79 HDO
A (d) 7.81
B (q) 4.22
C (t) 4.04
E (q) 1.80
F (m) 1.26
1.89
2.17
2.13
1.66
5.15
f1 (мд)

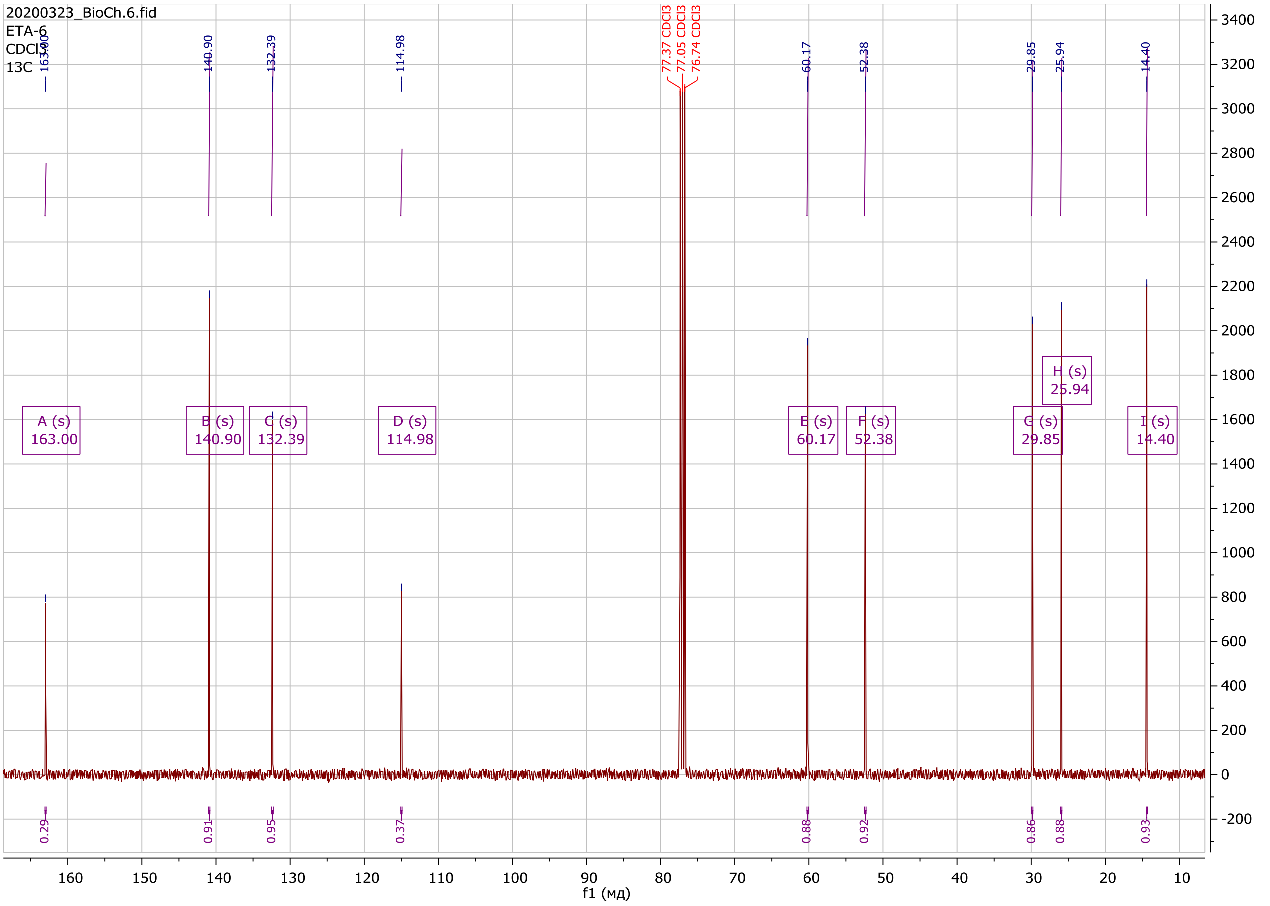

20200323_BioCh.6.fid
ETA-6
CDCl3
13C
77.37 CDCl3
77.05 CDCl3
76.74 CDCl3
A (s) 163.00
B (s) 140.90
C (s) 132.39
D (s) 114.98
E (s) 60.17
F (s) 52.38
G (s) 29.85
H (s) 25.94
I (s) 14.40
f1 (мд)

Compound 1g

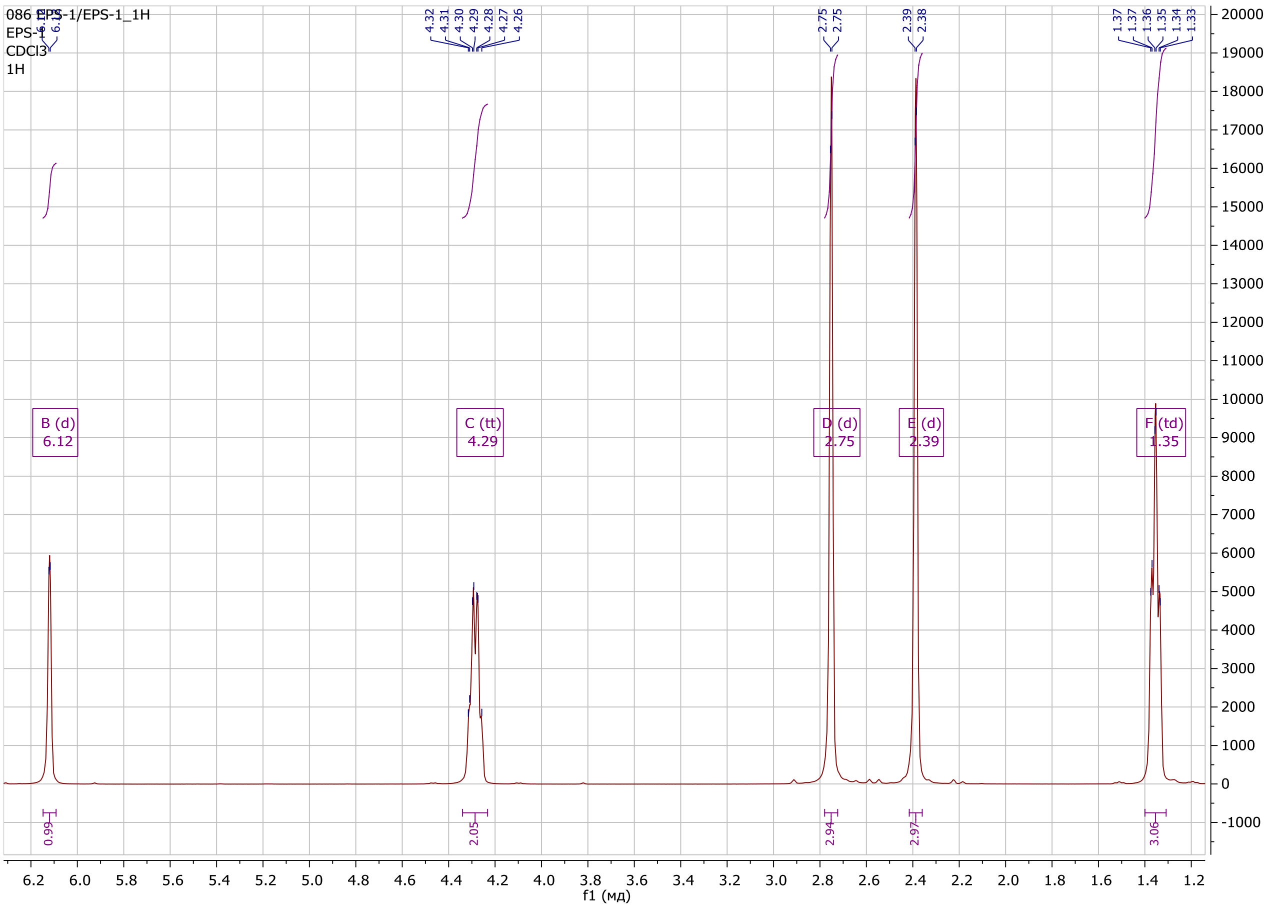
086 EPS-1/EPS-1_1H
EPS-1
CDCl3
1H
4.32
4.31
4.30
4.29
4.28
4.27
4.26
2.75
2.75
2.39
2.38
1.37
1.37
1.36
1.35
1.34
1.33
B (d)
6.12
C (tt)
4.29
D (d)
2.75
E (d)
2.39
F (td)
1.35
0.99
2.05
2.94
2.97
3.06
f1 (мд)

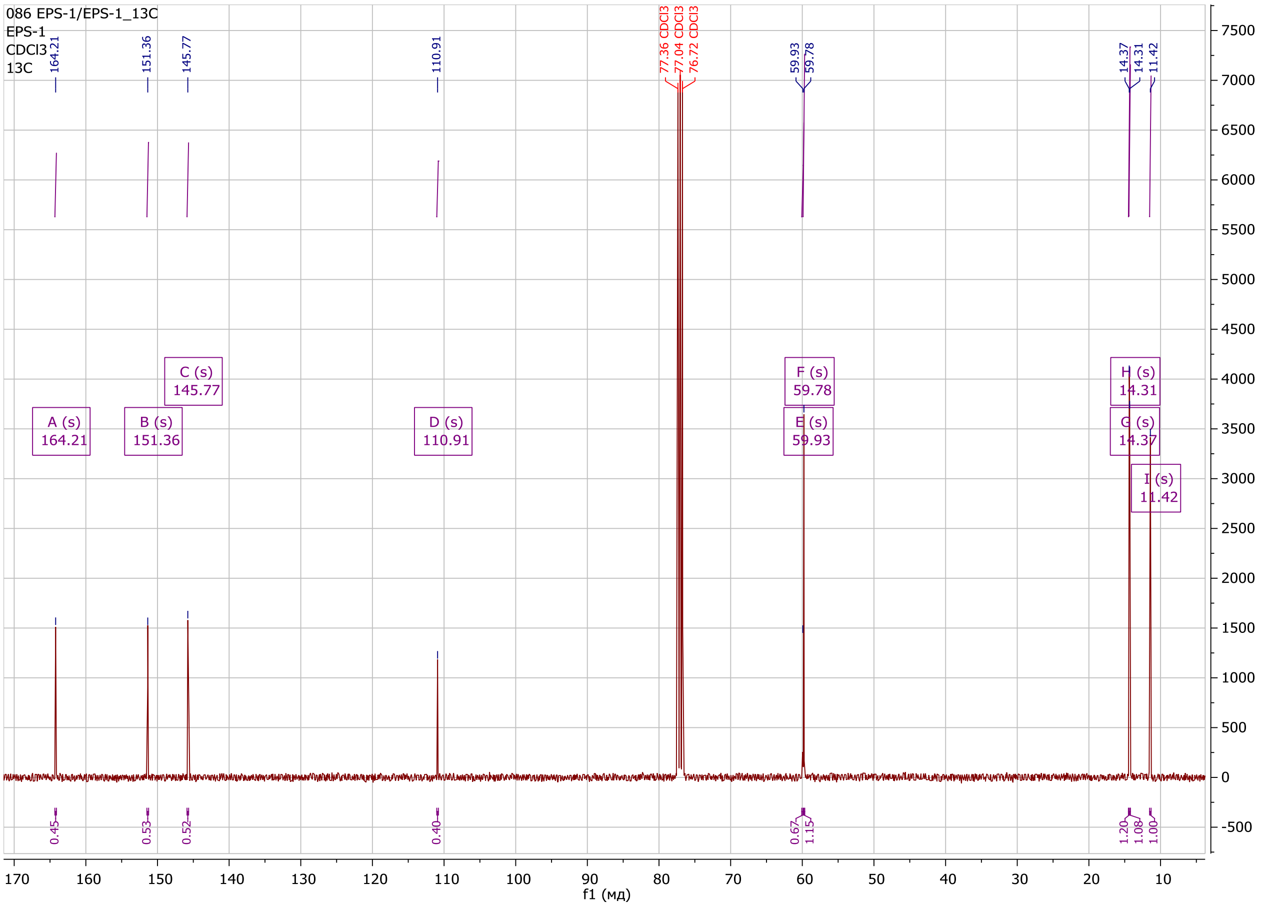
086 EPS-1/EPS-1_13C
EPS-1
CDCl3
13C
164.21
151.36
145.77
110.91
77.36 CDCl3
77.04 CDCl3
76.72 CDCl3
59.93
59.78
14.37
14.31
11.42
A (s)
164.21
B (s)
151.36
C (s)
145.77
D (s)
110.91
F (s)
59.78
E (s)
59.93
H (s)
14.31
G (s)
14.37
I (s)
11.42
0.45
0.53
0.52
0.40
0.67
1.15
1.20
1.08
1.00
f1 (мд)

Compound 3g

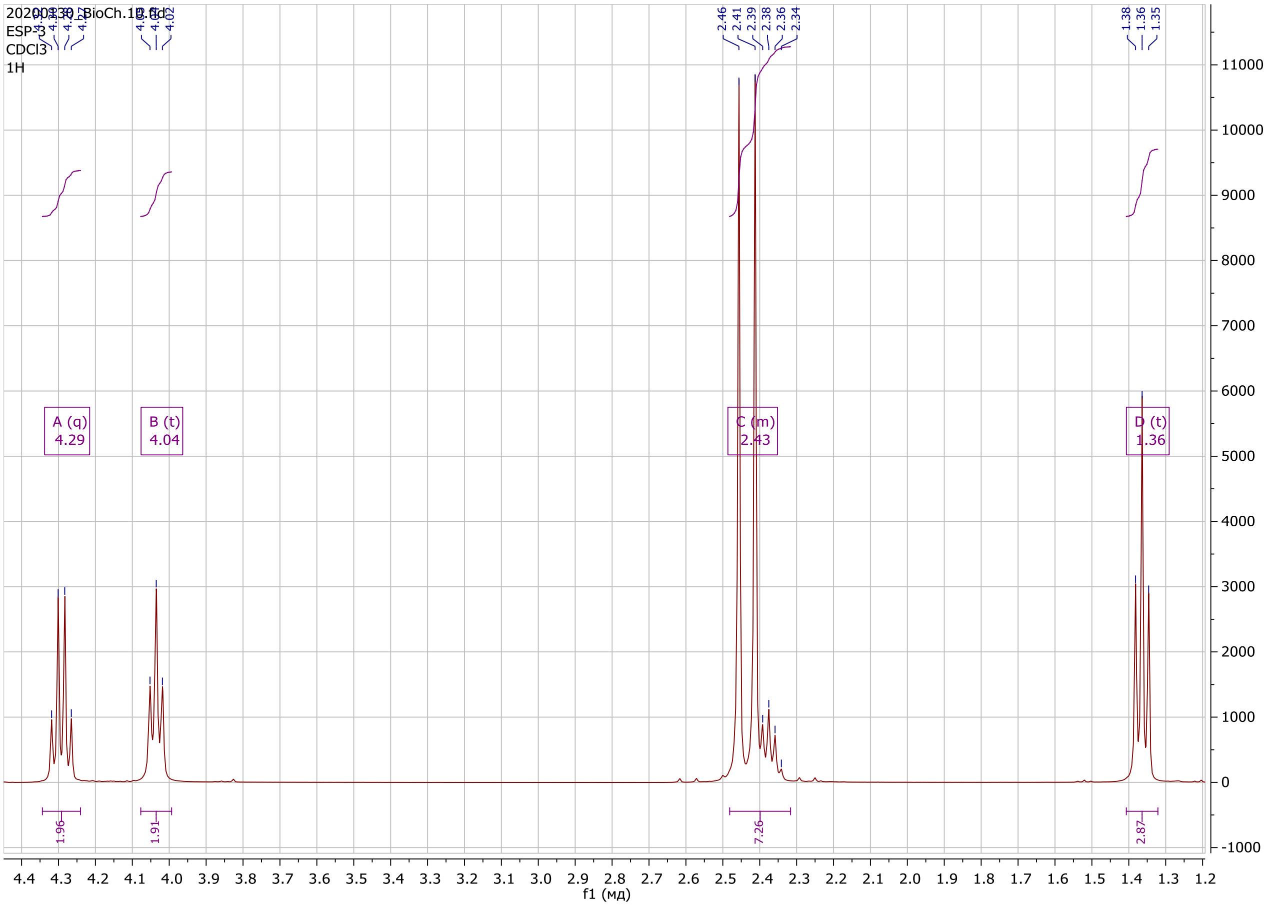

20200130_BioCh.10.fid
ESP-3
CDCl3
1H
A (q)
4.29
B (t)
4.04
C (m)
2.43
D (t)
1.36
1.96
1.91
7.26
2.87
f1 (мд)

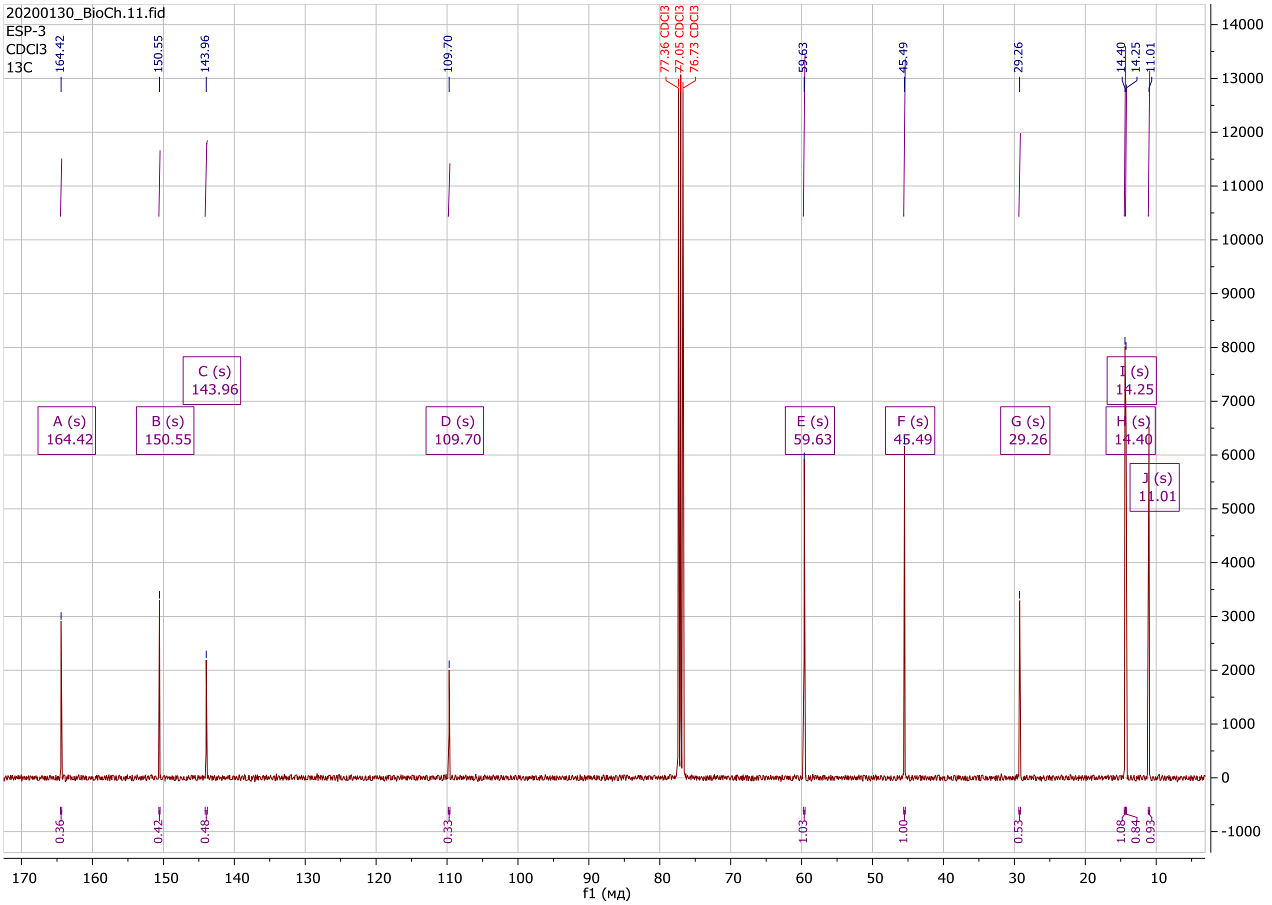

20200130_BioCh.11.fid
ESP-3
CDCl3
13C
77.36 CDCl3
77.05 CDCl3
76.73 CDCl3
A (s)
164.42
B (s)
150.55
C (s)
143.96
D (s)
109.70
E (s)
59.63
F (s)
45.49
G (s)
29.26
H (s)
14.40
I (s)
14.25
J (s)
11.01
f1 (мд)

Compound 4g

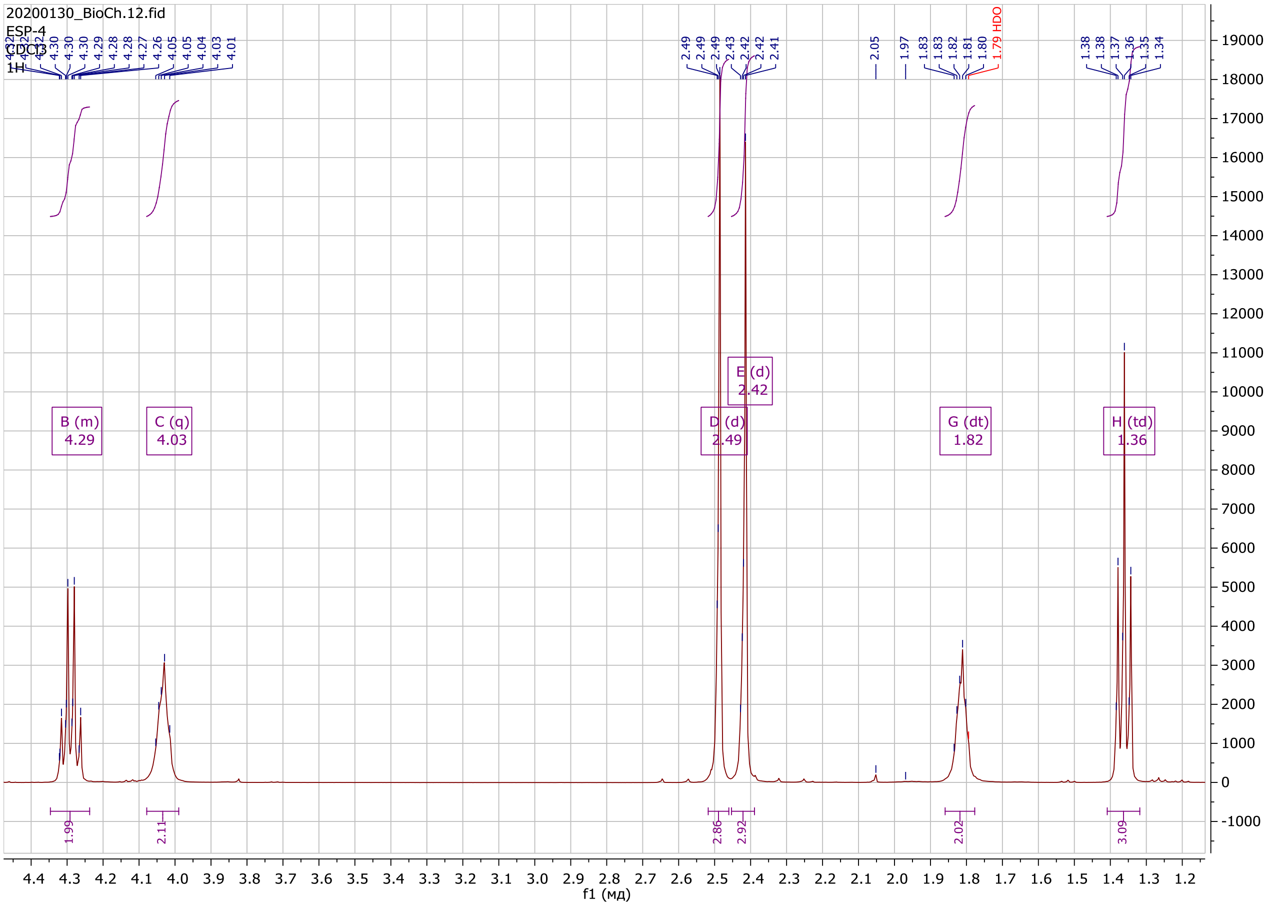
20200130_BioCh.12.fid
ESP-4
CDCl3
1H
1.79 HDO
B (m) 4.29
C (q) 4.03
D (d) 2.49
E (d) 2.42
G (dt) 1.82
H (td) 1.36
1.99
2.11
2.86
2.92
2.02
3.09
f1 (мд)

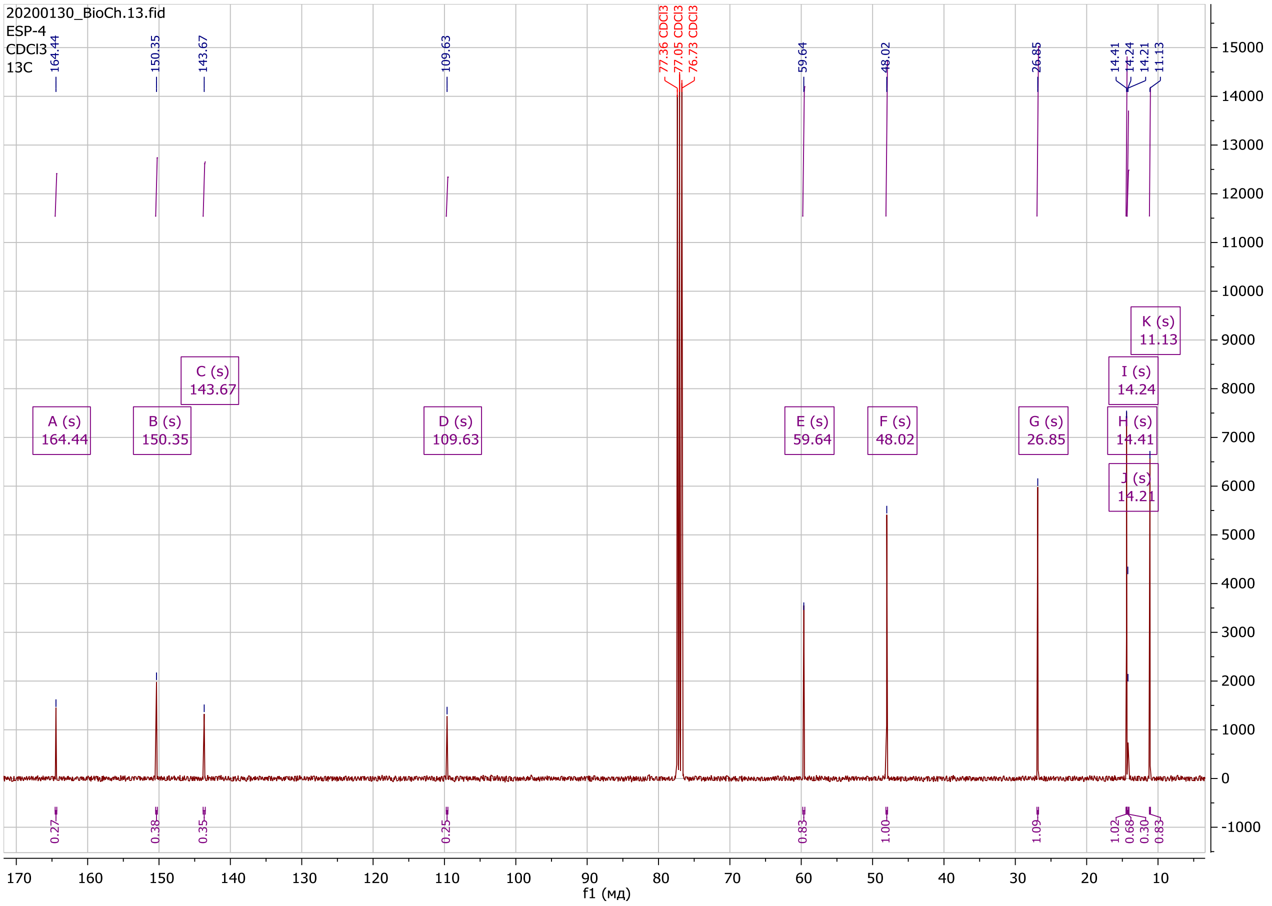
20200130_BioCh.13.fid
ESP-4
CDCl3
13C
77.36 CDCl3
77.05 CDCl3
76.73 CDCl3
A (s) 164.44
B (s) 150.35
C (s) 143.67
D (s) 109.63
E (s) 59.64
F (s) 48.02
G (s) 26.85
H (s) 14.41
I (s) 14.24
J (s) 14.21
K (s) 11.13
f1 (мд)

Compound 5g

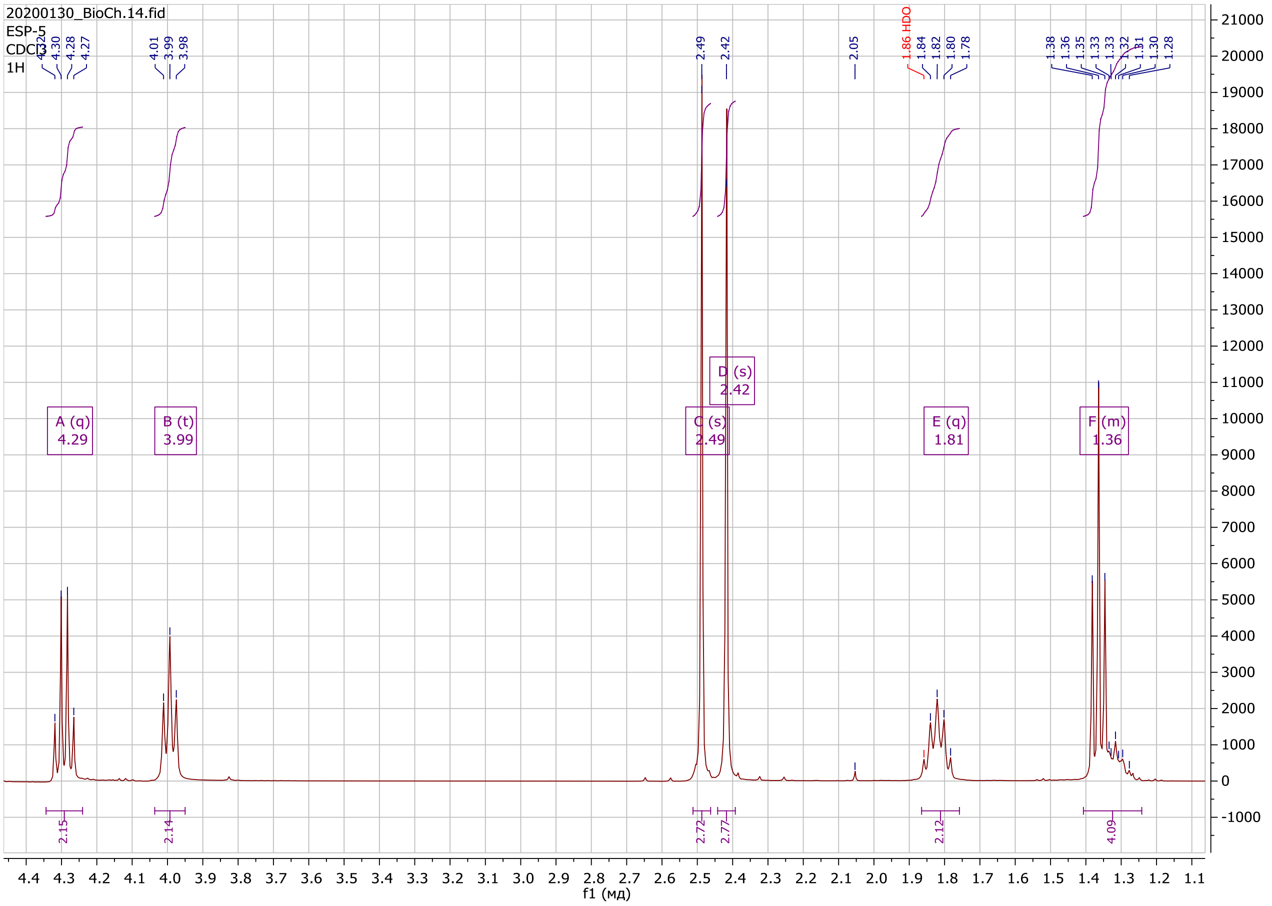

20200130_BioCh.14.fid
ESP-5
CDCl3
1H
A (q) 4.29
B (t) 3.99
C (s) 2.49
D (s) 2.42
E (q) 1.81
F (m) 1.36
1.86 HDO
2.15
2.14
2.72
2.77
2.12
4.09
f1 (мд)

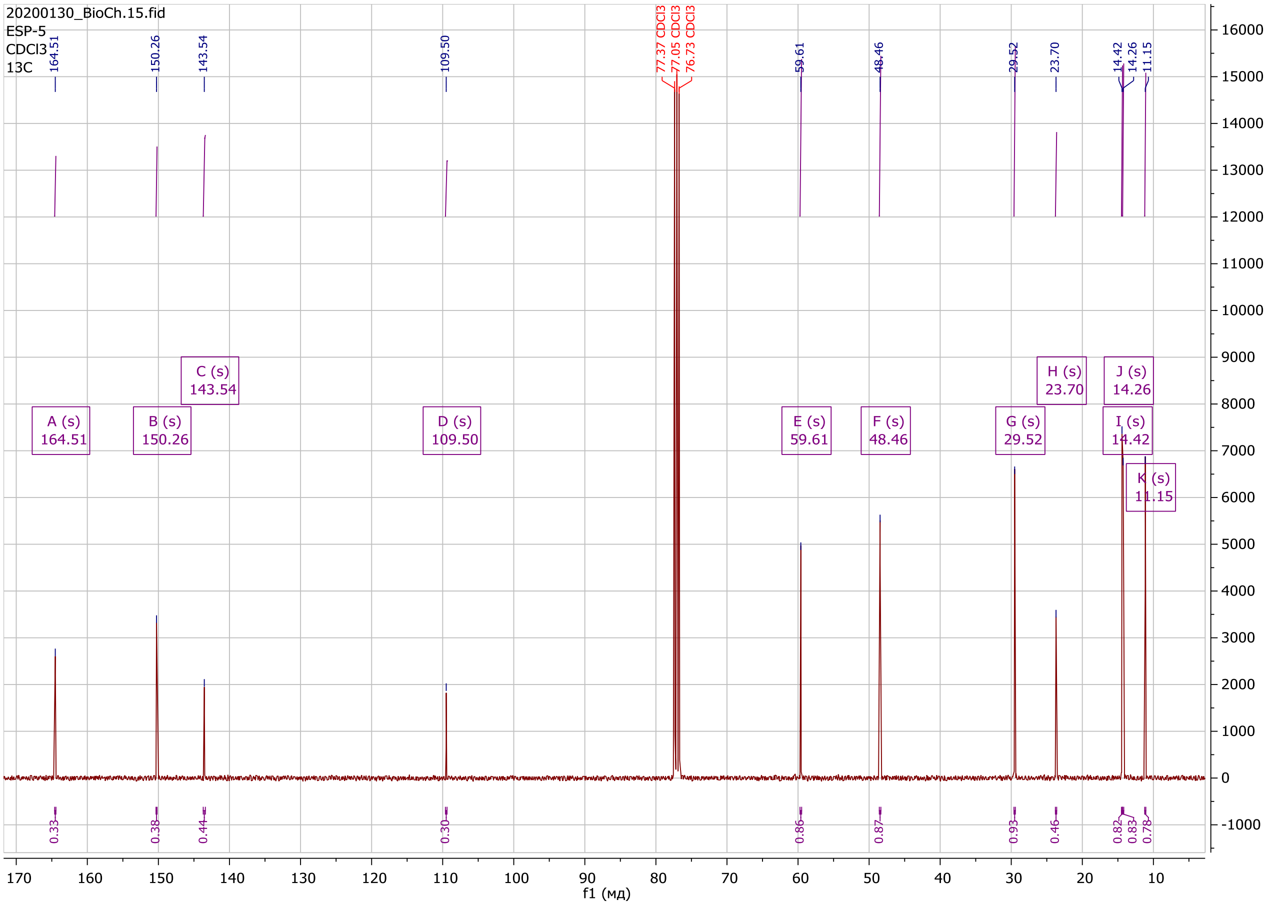

20200130_BioCh.15.fid
ESP-5
CDCl3
13C
77.37 CDCl3
77.05 CDCl3
76.73 CDCl3
A (s) 164.51
B (s) 150.26
C (s) 143.54
D (s) 109.50
E (s) 59.61
F (s) 48.46
G (s) 29.52
H (s) 23.70
I (s) 14.42
J (s) 14.26
K (s) 11.15
f1 (мд)

Compound 6g

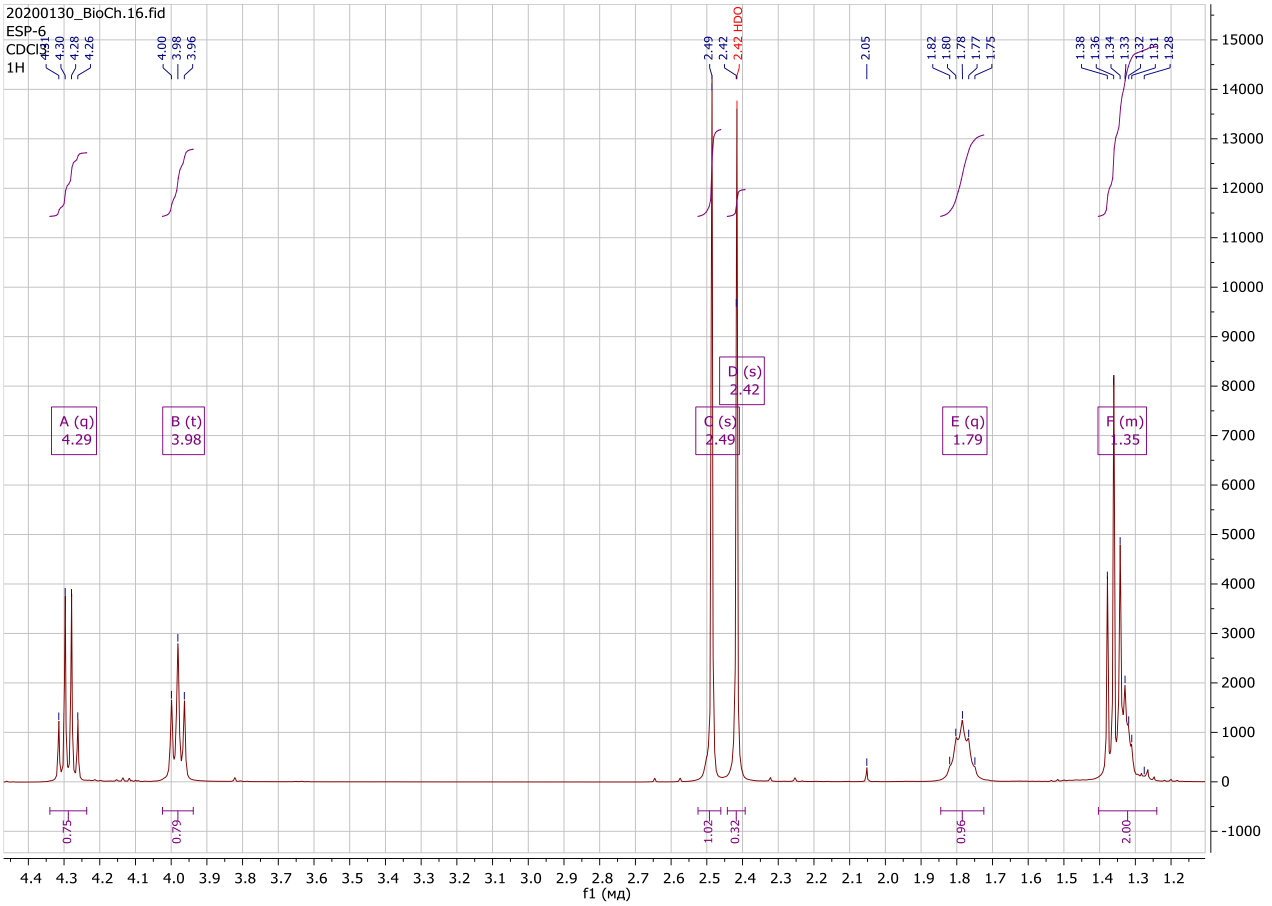
20200130_BioCh.16.fid
ESP-6
CDCl3
1H
4.31
4.30
4.28
4.26
4.00
3.98
3.96
2.49
2.42
2.42 HDO
2.05
1.82
1.80
1.78
1.77
1.75
1.38
1.36
1.34
1.33
1.32
1.31
1.28
A (q) 4.29
B (t) 3.98
C (s) 2.49
D (s) 2.42
E (q) 1.79
F (m) 1.35
0.75
0.79
1.02
0.32
0.96
2.00
f1 (мд)

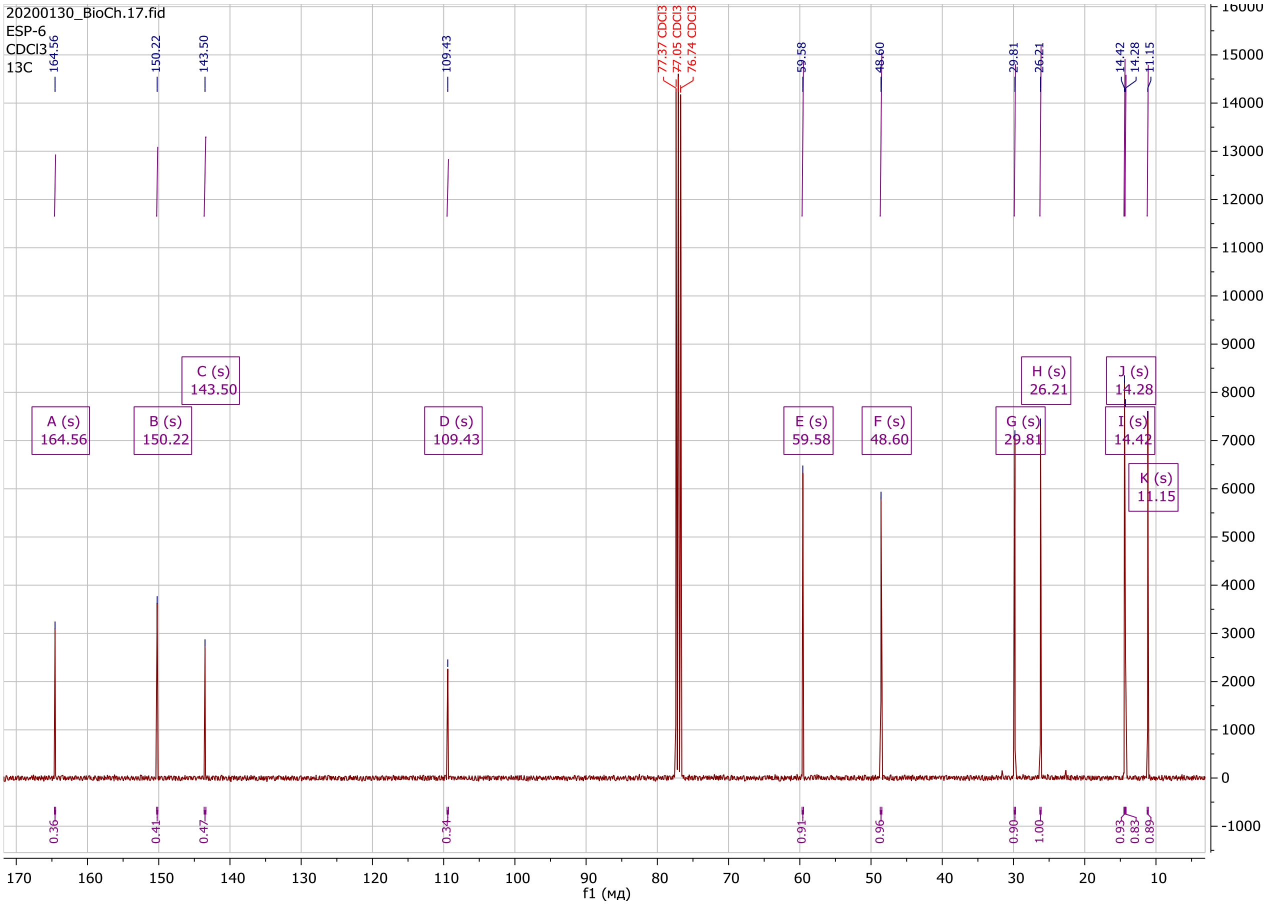
20200130_BioCh.17.fid
ESP-6
CDCl3
13C
164.56
150.22
143.50
109.43
77.37 CDCl3
77.05 CDCl3
76.74 CDCl3
59.58
48.60
29.81
26.21
14.42
14.28
11.15
A (s) 164.56
B (s) 150.22
C (s) 143.50
D (s) 109.43
E (s) 59.58
F (s) 48.60
G (s) 29.81
H (s) 26.21
I (s) 14.42
J (s) 14.28
K (s) 11.15
0.36
0.41
0.47
0.34
0.91
0.96
0.90
1.00
0.93
0.83
0.89
f1 (мд)
